# Emerging integrated photonic neural network technologies for artificial intelligence: from devices to systems

**DOI:** 10.1093/nsr/nwag335

**Published:** 2026-06-13

**Authors:** Changhao Han, Qipeng Yang, Jun Qin, Mingjin Wang, Zhaozheng Yi, Mingxiao Li, Ye Tian, Shuhan Yue, Xueling Wei, Zenan Wu, Liuyan Han, Dechao Zhang, Jingxuan Chen, Haowen Shu, Xingjun Wang, Wanhua Zheng, John E Bowers

**Affiliations:** Department of Electrical and Computer Engineering, University of California, Santa Barbara, CA 93106, USA; State Key Laboratory of Photonics and Communications, School of Electronics, Peking University, Beijing 100871, China; Department of Fundamental Network Technology, China Mobile Research Institute, Beijing 100053, China; Center for Target Cognition Information Processing Science and Technology, Beijing Information Science and Technology University, Beijing 100192, China; Laboratory of Solid State Optoelectronics Information Technology, Institute of Semiconductors, CAS, Beijing 100083, China; Center of Materials Science and Optoelectronics Engineering, University of Chinese Academy of Sciences, Beijing 100049, China; Hangzhou Institute for Advanced Study, University of Chinese Academy of Sciences, Hangzhou 310024, China; Laboratory of Solid State Optoelectronics Information Technology, Institute of Semiconductors, CAS, Beijing 100083, China; Center of Materials Science and Optoelectronics Engineering, University of Chinese Academy of Sciences, Beijing 100049, China; Department of Electrical and Computer Engineering, University of California, Santa Barbara, CA 93106, USA; State Key Laboratory of Photonics and Communications, School of Electronics, Peking University, Beijing 100871, China; State Key Laboratory of Photonics and Communications, School of Electronics, Peking University, Beijing 100871, China; State Key Laboratory of Photonics and Communications, School of Electronics, Peking University, Beijing 100871, China; State Key Laboratory of Photonics and Communications, School of Electronics, Peking University, Beijing 100871, China; Department of Fundamental Network Technology, China Mobile Research Institute, Beijing 100053, China; Department of Fundamental Network Technology, China Mobile Research Institute, Beijing 100053, China; Laboratory of Solid State Optoelectronics Information Technology, Institute of Semiconductors, CAS, Beijing 100083, China; Center of Materials Science and Optoelectronics Engineering, University of Chinese Academy of Sciences, Beijing 100049, China; State Key Laboratory of Photonics and Communications, School of Electronics, Peking University, Beijing 100871, China; Frontiers Science Center for Nano-optoelectronics, Peking University, Beijing 100871, China; State Key Laboratory of Photonics and Communications, School of Electronics, Peking University, Beijing 100871, China; Frontiers Science Center for Nano-optoelectronics, Peking University, Beijing 100871, China; Peking University Yangtze Delta Institute of Optoelectronics, Nantong 226010, China; Laboratory of Solid State Optoelectronics Information Technology, Institute of Semiconductors, CAS, Beijing 100083, China; Center of Materials Science and Optoelectronics Engineering, University of Chinese Academy of Sciences, Beijing 100049, China; Hangzhou Institute for Advanced Study, University of Chinese Academy of Sciences, Hangzhou 310024, China; Department of Electrical and Computer Engineering, University of California, Santa Barbara, CA 93106, USA

**Keywords:** photonic neural networks, artificial intelligence, photonic computing, silicon photonics, optoelectronic integration

## Abstract

The rapid expansion of artificial intelligence (AI) has created an explosive demand for computing power, while traditional von Neumann-based electronic computing faces the memory and interconnect bottlenecks. By migrating critical operations to the optical domain, photonic neural networks (PNNs) offer a promising solution to significantly reduce system latency and energy consumption. Driven by silicon photonics, on-chip PNNs achieve high-density integration on scalable platforms, promoting the evolution of optoelectronic integration systems. This paper presents a comprehensive review of emerging integrated PNN technology for AI, employing a cross-layer framework that spans from fundamental physical devices to system-level implementation. Based on the requirements of PNNs, this review analyzes key hardware devices and mainstream computing core architectures, evaluates integration strategies and discusses advanced system packaging. Furthermore, the review provides a prospect on the field by identifying the remaining technical challenges and highlighting the transformative potential of PNNs as the infrastructure for next-generation AI.

## Teaser

Integrated photonic neural networks for AI are summarized from device foundations to computing architectures, extending toward system-level integration and advanced packaging, with key challenges ahead outlined.

## INTRODUCTION

The rapid growth of artificial intelligence (AI), particularly the breakthrough advancements in large language models (LLMs) and generative AI, has triggered an era of unprecedented demand for computing power, which is driven by the explosive growth in model parameter scales [1]. However, in the post-Moore era [2], confronted with continuously rising training and inference workloads, the underlying computing hardware infrastructure faces severe sustainability challenges, and the traditional von Neumann architecture, in which the processor and memory are physically separated and communicate through a shared bus, is increasingly constrained by the memory and interconnect limits [3]. Furthermore, as AI workloads surge, traditional electronic integrated circuits (EICs) struggle to maintain energy efficiency while delivering high throughput, eventually pushing against the physical boundaries of thermal power density [4]. Therefore, there is an urgent need for the industry to transform toward novel system architectures to break through these electronic bottlenecks, thereby providing a robust computing platform to support the continued advancement of AI [3].

In this context, emerging photonics technology has proven its superiority in long-distance

communication as well as short-reach data center interconnects by exploiting the characteristics of photons, including high bandwidth, high parallelism, low crosstalk and low loss [5,6]. Simultaneously, for computing tasks, leveraging the inherent advantages of photons to transfer key operations, such as matrix-vector multiplication (MVM), convolution operation and non-linear activation, from the electrical domain to the optical domain, photonic neural networks (PNNs) are expected to significantly reduce latency and energy consumption for computing systems [7–9]. While research on photonic computing systems originated in free-space optics [8], PNNs have shifted from free-space optics to photonic integrated circuits (PICs) with the maturity of integrated photonics in recent years, especially complementary metal oxide semiconductor (CMOS)-compatible silicon (Si) photonics technology [10], making the high-density integration on scalable optoelectronic fusion platforms possible [4]. Emerging integrated PNNs utilize PICs to implement large-scale parallel linear computing directly on chip scale, and further improve energy efficiency by combining this with the non-linear processing and storage capabilities of EICs at the system level. This transition from traditional pure electronic architectures to specialized optoelectronic integration AI engines is promising to break existing speed and power consumption barriers, providing ultra-high-speed, low-energy solutions for next-generation AI hardware.

Herein, this review aims to provide a comprehensive roadmap for emerging integrated PNN technology for AI across the entire chain, bridging the gap between fundamental physical devices to system-level implementation. Adopting a cross-layer framework, we begin by examining the hardware device foundations, detailing essential building blocks for signal generation, manipulation and detection, specifically analyzed from on-chip PNN system requirements. Building on this, we investigate photonic computing cores by analyzing several mainstream architectures, together with key implementations of optical non-linear activation functions. From a historical perspective, Fig. 1 demonstrates the evolution of photonic computing cores through several major PNN architectures along a visual development timeline, illustrating key milestones in this dynamic field. Then, we discuss material integration strategies, evaluating the progression of hybrid, heterogeneous and monolithic integration platforms. Furthermore, we explore system packaging as a key approach for practical deployment, tracing the path toward advanced functional architectures. Finally, we conclude with a prospect on the field, addressing remaining technical challenges and highlighting the transformative potential of PNNs as the next-generation AI hardware infrastructure.

**Figure 1.**
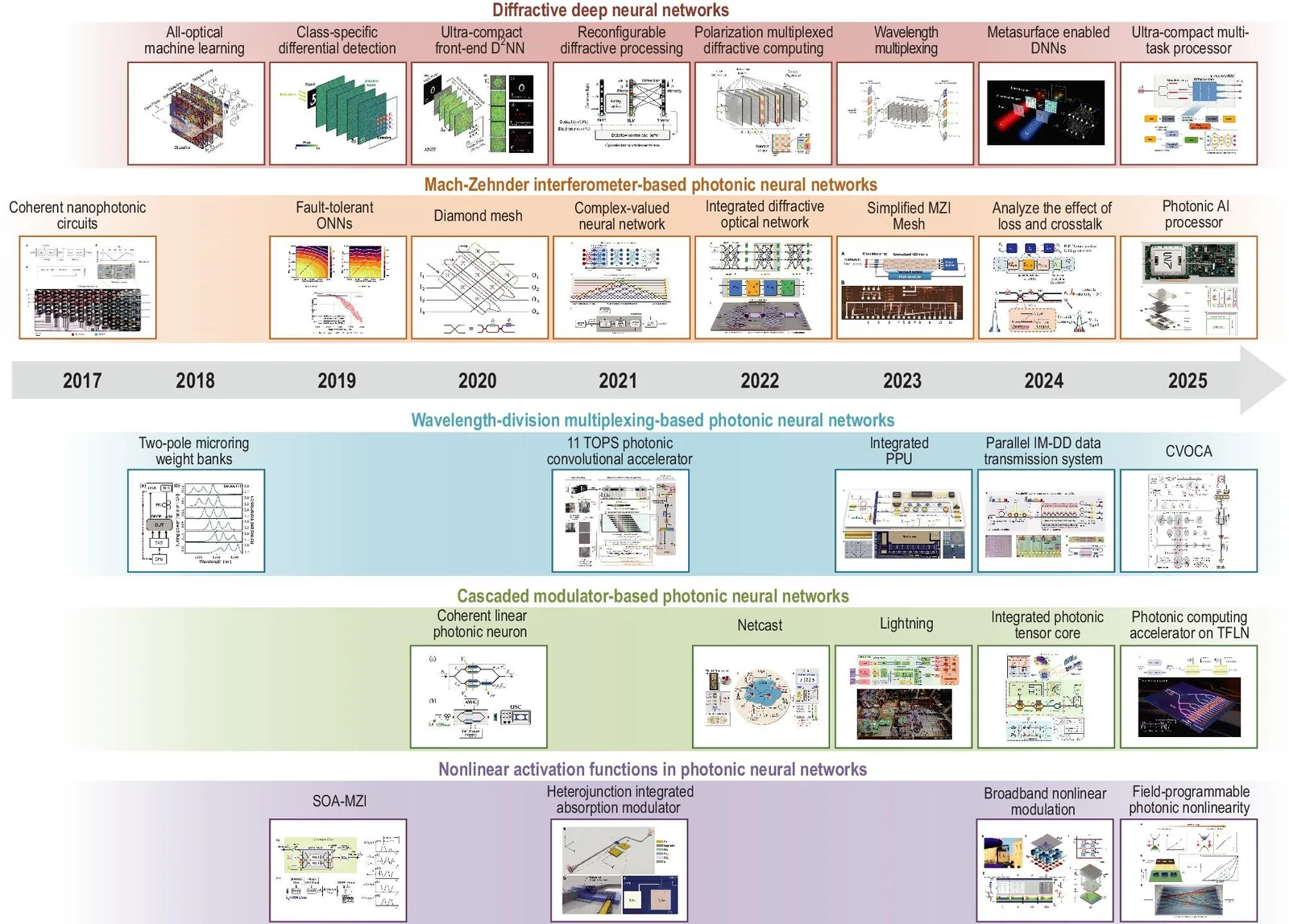
Evolution of photonic computing cores: a development timeline of several key PNN architectures. Diffractive deep neural networks (D^2^NNs): all-optical machine learning, reproduced with permission from Ref. [72], copyright: 2018 AAAS; class-specific differential detection, reproduced from Ref. [75] under a CC BY 4.0 license; ultra-compact front-end D^2^NN, reproduced with permission from Ref. [76], copyright: 2020 IEEE; reconfigurable diffractive processing, reproduced with permission from Ref. [85], copyright: 2021 Springer Nature; polarization multiplexed diffractive computing, reproduced from Ref. [82] under a CC BY 4.0 license; wavelength multiplexing, reproduced from Ref. [80] under a CC BY 4.0 license; metasurface enabled DNNs, reproduced with permission from Ref. [83], copyright: 2024 Wiley-VCH GmbH; ultra-compact multitask processor, reproduced from Ref. [86] under a CC BY 4.0 license. MZI-based PNNs: coherent nanophotonic circuits, reproduced with permission from Ref. [97], copyright: 2017 Springer Nature; fault-tolerant ONNs, reproduced from Ref. [104] under the OSA Open Access Publishing Agreement; diamond mesh, reproduced from Ref. [98] under the OSA Open Access Publishing Agreement; complex-valued neural network, reproduced from Ref. [101] under a CC BY 4.0 license; integrated diffractive optical network, reproduced from Ref. [99] under a CC BY 4.0 license; simplified MZI Mesh, reproduced from Ref. [100] under a CC BY 4.0 license; analyze the effect of loss and crosstalk, reproduced with permission from Ref. [105], copyright: 2024 IEEE; photonic AI processor, reproduced with permission from Ref. [108], copyright: 2025 Springer Nature. WDM-based PNNs: two-pole microring weight banks, reproduced with permission from Ref. [112], copyright: 2018 Optical Society of America; 11 TOPS photonic convolutional accelerator, reproduced with permission from Ref. [113], copyright: 2021 Springer Nature; integrated PPU, reproduced from Ref. [114] under a CC BY 4.0 license; parallel IM-DD data transmission system, reproduced from Ref. [126] under a CC BY 4.0 license; CVOCA, reproduced from Ref. [115] under a CC BY 4.0 license. CMod-based PNNs: coherent linear photonic neuron, reproduced with permission from Ref. [138], copyright: 2020 IEEE; netcast, reproduced with permission from Ref. [134], copyright: 2022 AAAS; Lightning, reproduced from Ref. [135] under a CC BY 4.0 license; integrated photonic tensor core, reproduced from Ref. [136] under a CC BY 4.0 license; photonic computing accelerator on TFLN, reproduced from Ref. [137] under a CC BY-NC-ND 4.0 license. Non-linear activation functions in PNNs: SOA-MZI, reproduced from Ref. [144] under the OSA Open Access Publishing Agreement; heterojunction integrated absorption modulator, reproduced from Ref. [145] under a CC BY 4.0 license; broadband non-linear modulation, reproduced from Ref. [146] under a CC BY 4.0 license; field-programmable photonic non-linearity, reproduced with permission from Ref. [151], copyright: 2025 Springer Nature.

## HARDWARE DEVICE FOUNDATIONS

Translating photonic computing models into high-performance PNN hardware requires the precise integration of photonic devices for signal generation, manipulation and detection, whose physical characteristics fundamentally determine the system’s bandwidth, density and energy efficiency. This section outlines the essential hardware device foundations for photonic computing, focusing on the specific requirements and recent advancements of core passive components and active devices including light sources, modulation units and detection interfaces to achieve scalable on-chip PNNs.

### Passive components

Passive photonic components, as fundamental building blocks of PNNs, leverage low-loss waveguide transmission and interferometric control to realize key functions such as signal routing, phase/amplitude modulation and matrix operations [7]. Among these, optical waveguides are the basic components to confine light within a core layer through the principles of total internal reflection or the photonic band gap [11]. Single-mode transmission within waveguides is crucial for computations in PNNs, while the presence of higher-order modes induces intermodal dispersion, resulting in signal distortion and substantially degrading interference efficiency, compromising the mathematical accuracy of matrix operations. For material platforms, Si-on-insulator (SOI) has a high refractive index contrast, which enables optical propagation through a tiny bending radius with negligible radiation loss, and this property facilitates a high integration density within SOI waveguides [11]. On the other hand, Si nitride (Si_3_N_4_) waveguides exhibit ultra-low transmission loss and a broad transparency window spanning from the visible to the mid-infrared [12], while they also can sustain high optical power densities due to the absence of two-photon absorption, essential for improving the dynamic range of PNNs. However, the material’s moderate refractive index contrast necessitates a relatively large bending radius, thereby limiting the Si_3_N_4_ device integration density [12].

In the construction of complex PNNs, optical waveguides require frequent crossings and handle intersections and delays. Traditional planar crossings often introduce crosstalk and insertion loss, which can severely limit network scalability. To address this, multimode interferences (MMIs) are employed to reduce insertion loss and suppress crosstalk, which are essential for enabling the cascading of large-scale matrices [6]. The MMI is a core device for signal distribution (broadcasting) and aggregation (summing), bridging external data and analog optical cores. Although originally used for power splitting, MMIs can also implement complex linear transformations, such as the discrete Fourier transform, and thus serve as optical convolution units in PNNs for efficient matrix multiplication [7]. In wavelength-division multiplexing (WDM) architectures, the MMI can act as a broadband convolution kernel. After optical signals encoding different characteristics are weighted by the modulator array in the wavelength domain, the N × 1 MMI acts as a broadband beam combiner to perform parallel summation over the inputs [6,8]. This function makes the MMI a key building block for neuronal fan-in and signal aggregation in PNNs.

The microring resonator (MRR) is a widely used passive component in PICs [13]. With a compact footprint, strong wavelength selectivity and high tunability, it serves as a versatile building block for optical filters, wavelength routing and analog multiplication, enabling massive parallel processing through WDM and high-speed MVM [14]. The scalability and speed of MRR-based systems are governed by spectral properties: a larger free spectral range (FSR) allows more non-overlapping wavelength channels with reduced crosstalk, whereas the modulation speed is fundamentally limited by the photon lifetime in the cavity. Leveraging these physical characteristics, MRRs can selectively couple or filter specific wavelengths, making them indispensable for WDM processing units. Cascading multiple MRRs allows for the creation of independent transmission windows at distinct wavelengths, thereby enabling sophisticated signal selection, demultiplexing and routing operations. In MVM operations, each matrix element maps to a continuously tunable weight carried by the MRR transmission [8]. By controlling the resonance shift via thermal or carrier modulation, the transmission can be continuously adjusted between these extrema, physically realizing analog weight assignment in the interval [13]. For input signals carried by different wavelength channels, an MRR array can apply independent weights to multiple wavelength channels simultaneously before the weighted signals are multiplexed into a single waveguide. Overall, the MRR simultaneously acts as a tunable weighting unit and a multichannel parallel computing engine, making it a foundational module for computing architectures.

On the other hand, the Mach–Zehnder interferometer (MZI) is a fundamental and important passive device in PICs [6]. Unlike MRRs which operate on resonance principles, MZIs operate based on coherent interference, offering broad operational bandwidth and high thermal stability. For PNNs, the MZI is not merely an intensity modulator but serves as the elementary unit for performing linear unitary transformations. Due to its precise phase control capabilities and ease of cascade integration, the MZI has become the cornerstone for constructing large-scale PNNs and optical matrix multipliers. The scalability of MZI-based computing architectures relies on the theoretical foundation that any arbitrary *N* × *N* unitary matrix can be decomposed into a sequence of localized 2 × 2 rotations. Due to the precise linear tunability, MZI arrays have become the cornerstone for constructing large-scale PNNs and optical matrix multipliers [15]. By mapping high-dimensional MVM onto the parallel propagation of light, these systems achieve substantial gains in bandwidth and computational speed compared to electronic counterparts. Nevertheless, the deployment of large-scale MZI meshes faces physical constraints, primarily the relatively large footprint of the interferometric arms and the cumulative power consumption required for active phase stabilization [7]. These challenges necessitate advanced phase modulation mechanisms and closed-loop monitoring strategies to ensure high-precision inference.

The evolution of these passive devices has greatly enhanced the capability of PICs to process complex signal streams. This facilitates more efficient integration between photonic technology and electronic control circuitry, thereby harnessing the high bandwidth and low-latency advantages of photonics for hardware acceleration in AI applications.

### Light sources

For integrated PNNs, on-chip light sources are among the core devices determining system parallelism, coherence and scalability [16], which not only serve as the energy supply for PNNs but also constitute the core physical foundation determining computational precision, parallelism and system robustness.

Owing to their direct bandgap properties and mature epitaxial growth technologies, III–V compound semiconductors, particularly indium phosphide (InP)- and gallium arsenide (GaAs)-based platforms, have long served as the dominant material basis for semiconductor lasers. Among the available device architectures, edge-emitting distributed feedback lasers (DFBs) and distributed Bragg reflector lasers (DBRs), implemented on these platforms, have emerged as practical on-chip light-source candidates for integrated photonic computing systems because of their stable single-frequency operation, wavelength selectivity and compatibility with mature semiconductor fabrication processes [16]. Within this category, continuous advances in cavity engineering have further improved device performance, as exemplified by gain-coupled DFB lasers [17] and transverse-photonic-crystal-based narrow-linewidth high-power lasers [18], which jointly enhance efficiency, output power and single-mode stability. In addition, the choice of gain medium plays a critical role: compared with conventional quantum-well (QW) lasers, quantum-dot (QD) DFB/DBR lasers typically exhibit a lower linewidth enhancement factor, reduced chirp and phase noise and improved temperature robustness, thereby facilitating the realization of single-frequency, narrow-linewidth sources with low relative intensity noise and enhanced tolerance to optical feedback [19]. Beyond edge-emitting lasers, vertical-cavity surface-emitting lasers (VCSELs) represent another important class of on-chip sources, distinguished by their low threshold current, high-speed direct modulation capability and suitability for dense two-dimensional (2D) integration, which make them attractive for high-throughput signaling and scalable PNN implementations [20].

The evolution of PIC platforms has enabled tighter integration of III–V gain materials with low-loss passive waveguide circuits, thereby supporting the realization of high-performance chip-scale lasers. By incorporating external feedback structures with extended cavity lengths and ultra-low loss, external cavity lasers (ECLs) can suppress laser linewidth and phase noise while improving operational robustness. Hybrid-integrated ECLs can be realized by co-packaging a III–V gain chip with a PIC-based external-cavity chip. Semiconductor lasers with hertz-level linewidths achieved through self-injection locking to CMOS-ready ultra-high-Q MRRs have demonstrated the low-noise potential and manufacturability of this approach [21]. In packaged interconnect environments, where optical reflection and feedback are often unavoidable, sub-hertz-linewidth, isolator-free hybrid-integrated ECLs combine excellent coherence with strong feedback tolerance [22], making them attractive for chip-level computing systems. However, the physical separation between these two components results in a substantial system footprint and limited integration density. To overcome these constraints, the heterogeneous integration of III–V materials onto PIC platforms has emerged as a critical research frontier for on-chip light sources, with primary technical routes including wafer bonding, flip-chip bonding, microtransfer printing and monolithic heteroepitaxy. Among these approaches, III–V gain has been integrated with Si photonics through bonding and related three-dimensional (3D) integration processes. When combined with low-loss Si_3_N_4_ external-cavity and filtering platforms, this strategy has enabled high-power lasers with sub-kilohertz linewidths on Si [23,24]. Representatively, the structure of the ultra-low-noise isolator-free heterogeneously integrated III–V/Si/Si_3_N_4_ laser is demonstrated in Fig. 2a [23]. In parallel, microtransfer printing and flip-chip bonding offer a scalable manufacturing route for on-chip integration of multiple devices, enabling the replicable integration of III–V-on-Si_3_N_4_ amplifiers/lasers, which further supports widely tunable narrow-linewidth ECLs [25]. Additionally, the monolithic InP/SOI platform presents another scheme where active III–V components and SOI circuits are combined within a single process stack [26].

**Figure 2.**
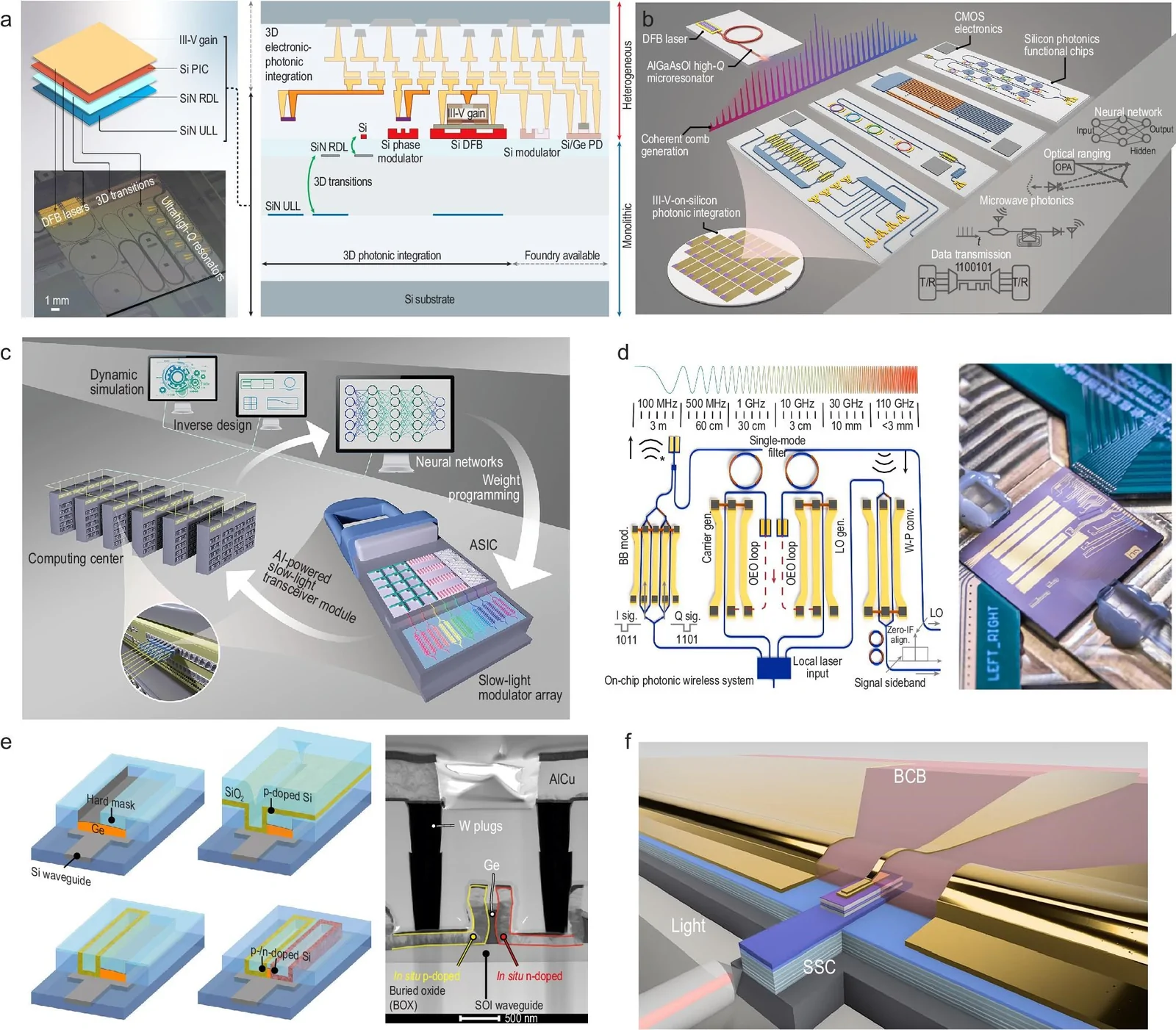
Representative core active devices for PNNs. (a) Ultra-low-noise isolator-free heterogeneously integrated III–V/Si/Si_3_N_4_ laser. Reproduced from Ref. [23] under a CC BY 4.0 license. (b) Integrated microcomb-driven Si photonics systems for different applications. Reproduced from Ref. [32] under a CC BY 4.0 license. (c) AI-accelerated Si photonic technology concept based on Si slow-light modulators. Reproduced from Ref. [39] under a CC BY 4.0 license. (d) Ultra-broadband on-chip photonics system for full-spectrum wireless communications based on TFLN modulators. Reproduced from Ref. [43] under a CC BY 4.0 license. (e) Process flow and design for the ultra-high-speed GeSi/Si PD. Reproduced from Ref. [50] under a CC BY 4.0 license. (f) Waveguide-integrated PD based on InP/InGaAs epitaxy with ultra-high bandwidth and high external responsivity. Reproduced with permission from Ref. [52]. Copyright: 2025 Springer Nature.

While the above approaches mainly optimize the coherence, linewidth and integration density of individual light sources, large-scale PNNs additionally require source multiplexing in the spatial and spectral domains to support massively parallel computation. In large-scale PNN architectures, high-dimensional parallel computing is essential to meet the demands of massive data throughput and complex MVM. Consequently, beyond optimizing the spectral purity and frequency stability of individual laser sources, the exploitation of spatial and spectral degrees of freedom is required to expand computational capacity. Multiwavelength integrated laser arrays provide a robust foundation for parallelized on-chip convolutional processing [27], while VCSELs, characterized by their intrinsic potential for dense two-dimensional integration, offer a viable path for spatial-temporal multiplexed PNN systems [28]. Furthermore, to address stringent constraints on footprint, power density and phase coherence in large-scale systems, optical frequency combs (OFCs) generated from a single cavity offer an attractive alternative to discrete laser arrays and can alleviate integration bottlenecks [29–31]. By combining an integrated OFC source with Si PICs, several microcomb-driven photonics systems including PNNs, optical interconnections, microwave photonics and optical ranging can be realized, as shown in Fig. 2b [32]. Regarding OFCs, mode-locked laser combs (MLL-combs), Kerr microresonator combs (Kerr-combs) and electro-optic modulation combs (EOM-combs) represent three primary implementation paradigms. MLL-combs exhibit high interline coherence and low phase noise, serving as high-precision multifrequency carriers for coherent PNNs and phase-sensitive computational operations. Nevertheless, their practical implementation is constrained by stringent mode-locking requirements and environmental sensitivity, necessitating integrated architectures and packaging strategies that incorporate active locking, thermal management and drift compensation [31]. Kerr-combs align closely with the scalability requirements of PNNs, as a single source can generate hundreds of equidistant spectral lines to enable WDM parallelism without the need for discrete laser arrays. This capability has supported numerous demonstrations of high-throughput convolutional and tensor-core-based photonic computing architectures [29,30]. Despite these advantages, spectral uniformity, per-line power and long-term stability remain pivotal challenges for field-deployable systems. Recent advancements in electrically pumped microcombs, achieved through the integration of III–V gain chips with Si_3_N_4_ MRRs via self-injection locking, have substantially increased on-chip power and the number of usable spectral lines [33]. Such developments demonstrate improved coherence metrics, including intrinsic linewidth, which facilitate the transition of microcombs from laboratory demonstrations to deployable PNN light sources. Furthermore, the spectral spacing of EOM-combs is governed by the driving microwave frequency, offering high reconfigurability and simplifying frequency-grid synchronization and drift tracking [31]. Established architectures, including microring and resonator-enhanced EOM-combs, continue to evolve toward higher efficiency and broader bandwidths, further maturing the landscape of on-chip microcomb sources.

### Modulation units

In integrated PNN architectures, electro-optic modulators (EOMs) serve as the critical bridge between the electronic and photonic signal domains [6,34]. Their core function is to convert electrical signals produced by digital circuits, which represent neural network weights or input activations, into optical signals in real time with high efficiency by modulating the amplitude or phase of the optical carrier [35]. As the input stage of PNNs, the EOM’s bandwidth determines the system’s inference speed, while its insertion loss and required drive voltage directly constrain the overall energy efficiency budget. Simultaneously, the EOM and radio frequency (RF) amplifier need to be designed collaboratively [36]. The EOM maps electrical signals to the load characteristics of optical amplitude or phase, while the RF amplifier determines the available swing, source impedance, rise/fall time and noise. However, these figures of merit are interdependent and exhibit pronounced trade-offs, thus device optimization needs to be tailored to the specific PNN applications.

At present, optoelectronic platforms are mainly based on Si photonics integration technology [10]. Based on Si photonic platforms, EOMs can be categorized into pure Si EOMs and Si‑based heterogeneous EOMs, by whether external EO functional materials are introduced on SOI substrates [16]. Pure Si EOMs rely on the plasma dispersion effect in Si waveguides, offering excellent CMOS-process compatibility for low-cost and high‑volume manufacturability, with co‑integration feasibility with microelectronic devices [35,37]. Among different electrical structures, the carrier‑depletion (reverse‑biased p-type/n-type semiconductor junction (PN junction)) approach offers the most balanced performance, while also boasting the highest engineering feasibility and application viability [34]. Depletion-mode Si EOMs tune the index by widening the depletion region under reverse bias, with high bandwidth, fast response, strong mode overlap and process maturity, and remain the mainstream approach in practical PNN applications. For non-resonator structures, Si Mach–Zehnder modulators (MZMs) inherently possess large bandwidth and high linearity due to their interferometric mechanism, making them crucial in PNNs that require high-precision linear operators, and they also offer advantages such as good thermal stability, high extinction ratio, wide operating wavelength range, low chirp and insensitivity to process variations [34–36]. Currently, conventional Si depletion-type MZMs typically achieve a 3 dB EO bandwidth of 30–60 GHz with a modulation efficiency (*V*π·*L*) of ∼1–2 V cm [6,34]. Nevertheless, Si MZMs often require millimeter‑scale phase‑shifter lengths, causing a footprint mismatch with microelectronic devices, while the optical loss and energy consumption (e.g. termination and amplifier power) also remain relatively high. For resonator structures, Si microring modulators (MRMs) achieve modulation by exploiting the coupling between a doped MRR and a straight waveguide [13]. Through this approach, the modulation efficiency can be enhanced effectively (<1 V cm), and typical ring radius is on the order of a few to a few tens of microns [6,13,34,38]. The junction capacitance of MRMs is small, and the energy per switching can be reduced to the fj/bit level with a drive swing below 1 V. These advantages significantly increase the integration density of PNNs. However, due to limitations imposed by the photon lifetime, Si MRMs possess a narrow operating wavelength window, typically <1 nm, making them highly temperature‑sensitive [6,13,34]. Therefore, when scaling to PNN arrays, the accumulated static thermal power consumption can become a system bottleneck. Meanwhile, at the system level, some recent works have been demonstrated the intersection of AI and Si EOMs based on CMOS-compatible pure Si platforms, achieving ultra-high-speed transmission of single-lane 400 Gbps based on compact Si slow-light modulators [39,40], as illustrated in Fig. 2c, which has brought new inspiration for the future optoelectronic integration of on-chip photonic interconnection and computing systems.

Pure Si EOMs offer decisive advantages in cost and CMOS-process compatibility, yet their limited plasma dispersion effect constrains speed and energy efficiency. To overcome these bottlenecks, heterogeneously integrating materials with superior EO performance on Si has become an effective approach to simultaneously boost bandwidth and efficiency. For Pockels effect platforms, thin-film lithium niobate (TFLN), with a pronounced Pockels coefficient (*r*_33_ ∼ 30 pm/V), exhibits excellent linearity and ultra-wide bandwidth [41]. In recent years, traveling-wave MZMs based on TFLN have consistently achieved EO bandwidths above 100 GHz while maintaining low intrinsic optical loss and good linearity [41,42], making them suitable for microwave photonics and PNNs that require analog-domain precise accumulation and are highly sensitive to distortion. Representatively, based on TFLN MZMs, an ultra-broadband on-chip photonics system for full-spectrum wireless communications is demonstrated recently [43], as shown in Fig. 2d, which enables full-link wireless communication across nine consecutive bands and speeds up to 100 Gbps. However, mainstream TFLN MZMs still exhibit low modulation efficiencies, requiring millimeter-scale phase-shifter arms and high-quality traveling-wave electrodes to meet speed, impedance and velocity-matching requirements, which increases footprint and RF design complexity and lowers integration density [41]. Accordingly, they are better suited to medium-to-large-scale photonic computing cores that prioritize linearity and signal-to-noise ratio (SNR) performance. On the other hand, coupling organic EO polymers to Si waveguides can also deliver a higher effective EO coefficient and improved modulation efficiency, but challenges regarding poling stability, thermal aging and long-term reliability still limit their industrial-scale production for PNN deployment [44]. Along the electro-absorption modulator (EAM) path based on the Franz–Keldysh effect and the quantum-confined stark effect, germanium (Ge)/Si [45] and III–V/Si [46] can realize compact EAMs on Si substrates, which are typically tens to hundreds of microns in length, with small capacitance, low drive voltage and high EO bandwidth; however, their amplitude modulation exhibits chirp, increasing the requirements for link equalization in PNNs, and the lattice mismatch between Ge or III–V materials and Si substrates imposes limitations on yield and cost [34]. Overall, heterogeneous integration extends the device performance frontier by incorporating high-EO coefficient materials. By introducing heterogeneous functional materials on low-cost Si substrates, Si-based heterogeneous EOMs substantially expand the trade-off space among bandwidth, efficiency, energy consumption and linearity, enabling high integration and high-speed transmission while preserving cost and scalability, and providing new options for high-performance PNN modulation units.

### Photodetection interfaces

As the core interfaces for converting optical signals to electronic bytes, the performance of photodetectors (PDs) defines the operational throughput and inference precision of PNNs from the physical foundation upward [47]. In many integrated PNN architectures, PDs serve functions that extend beyond terminal readout. In addition to converting weighted optical signals into electrical outputs, they can support intermediate optical-to-electrical interfacing, electronics-assisted non-linear activation, error monitoring and on-chip calibration [48,49]. Accordingly, photodetection in PNNs places demands not only on responsivity and bandwidth, but also on noise level, linearity, dynamic range, dark current, array scalability and compatibility with front-end electronic circuitry. The overall readout performance is therefore shaped jointly by the detector material platform, device structure, detection architecture and co-design with electronic circuits such as transimpedance amplifiers (TIAs).

Among the integrated PD technologies considered for PNNs, Ge/SiGe and III–V PDs are among the most mature and widely discussed classes, although they are typically favored in different integration scenarios. Ge/SiGe PDs have become a major option for Si-based PNNs because they offer a favorable combination of CMOS compatibility, bandwidth and array scalability, making them well suited to large-scale Si photonic processors based on MZI meshes, MRR weight banks and WDM channels. State-of-the-art Ge/SiGe PDs have reached 3-dB bandwidths of up to 265 GHz, as shown in Fig. 2e, demonstrating that this platform can provide substantial intrinsic bandwidth for high-speed photonic readout [50]. For practical deployment, however, balanced device performance is often more important than record bandwidth alone. For example, Shi *et al*. reported a Ge/SiGe PD with a 103 GHz bandwidth, a responsivity of 0.95 A/W at 1550 nm, and a dark current of 1.3 nA through optimization of a U-shaped electrode geometry [51]. In large PD arrays, such low dark current can help suppress baseline offsets and reduce receiver noise contributions, which may in turn benefit system SNR and effective analog precision in PNN computation. Shi *et al*. also demonstrated a non-linear Ge–Si PD in which the transfer characteristic is shaped by the non-linear junction photoresponse associated with photocurrent compression, thereby illustrating the possibility of detector-level non-linear activation together with *in situ* monitoring and readout [47].

III–V PDs, in contrast, remain attractive for applications that require broad spectral coverage, low dark current or specific high-speed or gain-enabled functionalities; although their integration into large-scale CMOS-compatible photonic systems is generally more challenging than that of Ge/SiGe devices. Li *et al*., for example, decoupled the optical coupling direction from the carrier transport direction in an InP PD, achieving a 3-dB bandwidth of 206 GHz and an external responsivity of 0.81 A/W, as shown in Fig. 2f [52]. To improve manufacturability and facilitate co-integration with Si photonics, monolithic integration routes are also being actively explored. Mauthe *et al*. reported a monolithically integrated PIN PD with a responsivity of 0.68 A/W and an extremely small footprint [53], while Wen *et al*. demonstrated a monolithically integrated waveguide-coupled PIN PD with a dark current density of 0.048 A/cm² at −1 V, a responsivity of 0.2 A/W at −2 V, a cut-off frequency exceeding 70 GHz and 50 Gb/s reception capability [54]. Beyond these more established platforms, emerging approaches based on heterogeneous integration on TFLN [55], as well as graphene and other two-dimensional materials [56], are also attracting interest because of their potential for high-speed co-integration, broad spectral response and added functionality. However, at present, these approaches remain less mature for large-scale deployment than Ge/SiGe and III–V technologies.

Beyond the material platform itself, the appropriate photodetection scheme in a PNN is fundamentally determined by the physical quantity that must be recovered from the optical computation. In intensity-encoded PNNs, where outputs are represented primarily by optical power, direct detection is often preferred because it enables comparatively simple and energy-efficient optical-to-electrical conversion with relatively low receiver complexity [48,57]. In contrast, in architectures in which computation depends explicitly on optical phase, interference, coherent accumulation or complex-valued weights, direct detection is generally insufficient because it does not preserve the full optical field at the receiver. In such cases, coherent detection is often required to recover both amplitude and phase, typically by employing a local oscillator together with balanced or phase-sensitive receiver architectures [58,59]. This distinction also has important implications for integration complexity. Relative to direct detection, coherent readout imposes tighter requirements on waveguide loss, phase stability, local-oscillator distribution, detector matching, balanced receiver layout and alignment tolerance. Direct and coherent detection should therefore be regarded not simply as competing receiver options, but as distinct readout modalities matched to different classes of PNN architectures and computational formalisms.

In practical PNN receivers, front-end performance is determined not by PDs alone, but by the combined behavior of PDs, TIAs, parasitic elements and the overall readout architecture. PD–TIA co-design is therefore a key consideration for both fidelity and scalability [60,61]. For direct-detection PNNs, the front end must provide low noise and sufficiently linear power-to-voltage conversion over an adequate dynamic range while maintaining low power consumption and high channel density. For coherent PNNs, co-design becomes even more critical because sensitivity, common-mode rejection, channel matching, phase fidelity and balanced operation all depend strongly on the joint optimization of the PD pair and the TIA input stage. In both cases, junction capacitance, input impedance, thermal noise, shot noise, gain–bandwidth trade-offs and saturation behavior must be optimized collectively rather than in isolation. In large-scale analog PNNs, these receiver-level trade-offs can ultimately propagate to system-level metrics such as energy per multiply-accumulate (MAC) operation, area efficiency, calibration overhead and achievable effective analog precision. Photodetection in PNNs is therefore better viewed not as an isolated device problem, but as a co-designed optoelectronic interface whose performance emerges from the joint optimization of device physics, receiver topology and electronic readout circuitry.

### Non-volatile modulation and memory

The modulation mechanisms discussed above, whether plasma dispersion in Si, the Pockels effect in TFLN or electro-absorption in Ge/III–V, are inherently volatile: the optical state reverts once the driving signal is removed, mandating continuous electrical power to maintain programmed neural network weights. For PNNs targeting in-memory computing and large-scale weight storage, this imposes a substantial static power overhead that scales linearly with the number of programmable elements. Non-volatile photonic modulation, in which the optical state is retained without sustained electrical input, therefore represents a complementary and increasingly important device category for realizing energy-efficient, scalable PNNs. In 2023, Youngblood *et al*. provided a comprehensive summary of six major technology platforms for integrated optical memristors, including phase-change materials (PCMs), electronic memristors, charge-trapping devices, ferroelectric materials, micro-electro-mechanical systems (MEMS) and magneto-optic elements, benchmarking them across switching speed, energy, endurance, bit precision, extinction ratio and footprint [62].

PCMs constitute the most extensively investigated non-volatile photonic platform. PCMs undergo reversible structural transitions between amorphous and crystalline phases upon the application of optical or electrical pulses, producing a pronounced and stable refractive index contrast that can be leveraged for both amplitude and phase modulation on photonic waveguides. In 2019, Li *et al*. demonstrated over 5-bit non-volatile multilevel storage in a single Ge_2_Sb_2_Te_5_ (GST) photonic memory cell on an Si_3_N_4_ waveguide, using a double-step pulse scheme that enables state-independent single-pulse programming within 250 ns with an order-of-magnitude reduction in both switching time and energy relative to prior multipulse methods [63]. In the same year, Ríos *et al*. demonstrated in-memory scalar multiplication on a single integrated GST cell atop an Si_3_N_4_ waveguide by encoding operands in the device transmittance and input pulse energy, achieving linear, drift-free, non-volatile multilevel operation and establishing the foundation for scalable photonic matrix-vector architectures [64]. Earlier, in 2017, Cheng *et al*. realized an on-chip photonic synapse by placing discrete GST islands on a tapered Si_3_N_4_ waveguide, a geometry that yields a homogeneous evanescent field distribution and enables deterministic, cumulative synaptic weight programming solely by varying the number of identical fixed-energy optical pulses, with an exponential weight–pulse-number dependence naturally compatible with spike-timing-dependent plasticity [65].

A key limitation of conventional GST is its significant optical absorption in the crystalline state, which degrades insertion loss and constrains the achievable figure of merit (FoM) for phase-only modulation. To address this, in 2019, Zhang *et al*. introduced Ge_2_Sb_2_Se_4_Te_1_ (GSST), in which selenium substitution decouples refractive index modulation from optical loss, yielding an FoM more than two orders of magnitude above that of GST across the 1–18.5 μm range, and validated the material in a non-volatile Si_3_N_4_ MRR switch with 42 dB contrast ratio and sub-0.5 dB insertion loss [66]. From the standpoint of manufacturability and scalability, the integration of PCMs into commercial Si photonic foundries is critical. In 2024, Wei *et al*. demonstrated monolithic back-end-of-line integration of Sb_2_Se_3_ and GSST on 200 mm foundry-manufactured Si photonic wafers by introducing an Si_3_N_4_ etch-stop layer, achieving electrically programmable MZI switches, microring multilevel phase shifters exceeding 5-bit resolution and waveguide attenuators with over 7-bit intensity modulation [67]. In the same year, Chen *et al*. demonstrated deterministic quasi-continuous tuning of Sb_2_S_3_ integrated on a high-volume 300 mm Si photonic platform, further validating the scalability of PCM-based programmable PICs for large-scale deployment [68].

Beyond chalcogenide PCMs, ferroelectric materials offer an alternative non-volatile modulation pathway through electrically controllable domain reconfigurations that alter the effective Pockels coefficient. In 2022, Geler-Kremer *et al*. demonstrated a non-volatile multilevel photonic phase shifter based on epitaxial BaTiO_3_ integrated with Si waveguides, achieving eight distinct, reproducible and long-term-stable optical states with pure phase modulation, an insertion loss of only 0.07 dB and switching energies as low as 4.6 pJ [69]. In 2025, Li *et al*. reported optical memristors based on thin-film lead zirconate titanate fabricated via a wafer-scale sol–gel process, exploiting a functional duality governed by a threshold voltage: non-volatile refractive index setting through ferroelectric domain reconfiguration above the threshold, and ultra-fast volatile modulation via the Pockels effect below it, achieving a non-volatile index change of 4.6 × 10⁻^3^ with 12.3 pJ switching energy, endurance exceeding 10^5^ cycles and simultaneous support for 48 Gbps EO modulation [70].

Magneto-optic materials introduce a fundamentally distinct mechanism based on non-reciprocal refractive index modulation. In 2025, Pintus *et al*. demonstrated a magneto-optic memory cell by heterogeneously integrating cerium-substituted yttrium iron garnet (Ce:YIG) with Si MRRs, where an integrated electromagnet programs the magnetization of a CoFeB ferromagnetic layer to induce non-volatile, direction-dependent resonance shifts for counter-propagating optical modes [71]. This approach achieved a programming speed of 1 ns, a switching energy of 143 fJ per bit, multilevel encoding with 3.5-bit precision and sustained 2.4 × 10^9^ write–erase cycles without degradation, exceeding prior photonic memory endurance by over three orders of magnitude.

Overall, the benchmarking across these non-volatile platforms reveals that no single technology yet simultaneously optimizes switching speed, energy, endurance, bit precision, extinction ratio and footprint [62]. Key remaining bottlenecks include sub-10 ns bidirectional switching and high cycling endurance for electrically programmed PCM devices, as well as multibit non-volatile operation. Nevertheless, the rapid progress across these diverse material platforms underscores the strong potential of non-volatile photonic modulation as a critical enabler for next-generation energy-efficient PNNs.

## PHOTONIC COMPUTING CORES

Photonic computing cores serve as the central processing units of PNNs, where the actual MVM and non-linear operations take place. This section explores the diverse architectural paradigms used to implement these cores, covering diffractive deep neural networks (D^2^NN), PNNs based on MZI, WDM and cascaded modulators (CMods). Furthermore, the critical integration of non-linear activation functions is investigated, which are essential for enabling deep learning capabilities within these linear optical frameworks.

### D^2^NN

D^2^NN utilize the diffractive propagation of light to achieve photonic computing. From 2018 to 2025, the field advanced from qualitative validation to quantitative breakthroughs, establishing a comprehensive framework covering theoretical limits, robustness, multidimensional multiplexing and on-chip integration. The concept was pioneered by Ozcan’s group at UCLA in 2018. Leveraging the Huygens–Fresnel principle, they trained static terahertz diffractive layers to achieve light-speed, zero-energy MNIST classification with 91.75% blind testing accuracy and demonstrated an imaging lens function acting as an auto-encoder [72], whose fundamental framework is shown in Fig. 3a. This work defined the ‘computation by propagation paradigm, though it initially lacked a complete theoretical basis for system capacity.

**Figure 3.**
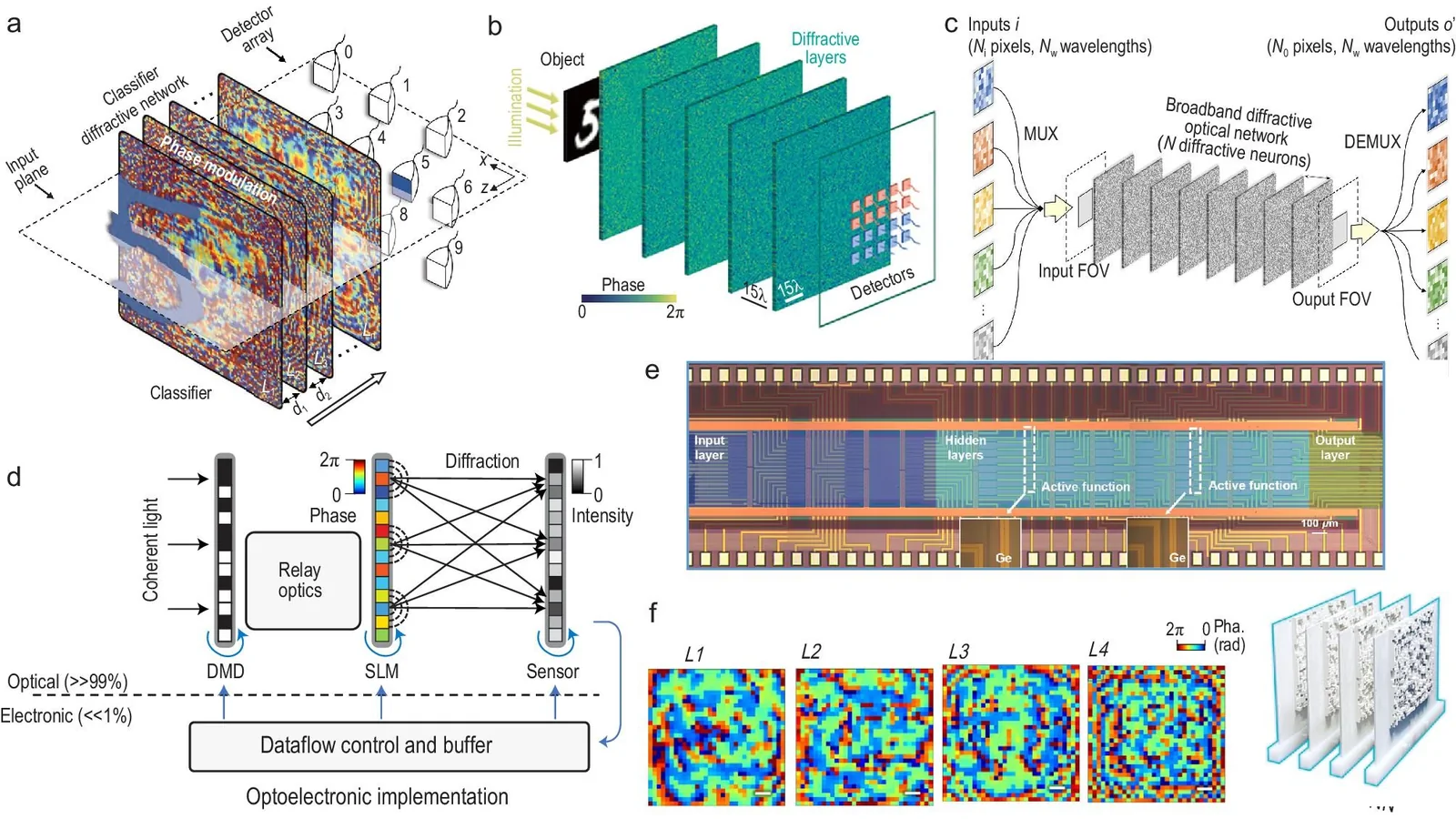
Architecture of D^2^NNs. (a) Fundamental D^2^NN framework. Reproduced with permission from Ref. [72]. Copyright: 2018 AAAS. (b) Class-specific differential detection. Reproduced from Ref. [75] under a CC BY 4.0 license. (c) WDM diffractive optical network. Reproduced from Ref. [80] under a CC BY 4.0 license. (d) Reconfigurable DPU. Reproduced with permission from Ref. [85]. Copyright: 2021 Springer Nature. (e) Highly integrated AON- D^2^NN. Reproduced from Ref. [87] under a CC BY 4.0 license. (f) Super-resolution diffractive neural network (S-DNN). Reproduced from Ref. [92] under a CC BY 4.0 license.

Subsequent research established the mathematical completeness of D^2^NNs. Kulce *et al*. in 2021 used singular value decomposition to prove that the solution space dimension scales linearly with layer count, validating the superiority of ‘deep’ over ‘wide’ networks [73]. In the same year, they derived the critical condition for universal computation: a multilayer system can synthesize arbitrary complex-valued linear transformations with near-zero error provided the number of diffractive neurons matches the product of input and output pixels [74].

To enhance practicality, Li *et al*. in 2019 introduced differential detection using positive–negative detector pairs to overcome the non-negativity of light intensity, boosting MNIST accuracy to 98.52% and CIFAR-10 accuracy to 50.82%, approaching electronic LeNet performance [75], utilizing class-specific differential detection as shown in Fig. 3b. Addressing hybrid computing, Mengu *et al*. in 2020 utilized D^2^NNs as front-end preprocessors to achieve a data compression ratio exceeding 7.8 times, allowing simplified backend electronics to maintain high precision [76].

Regarding robustness, Mengu *et al*. in 2020 proposed a ‘vaccination’ training strategy, which restored accuracy from <40% to >90% under mechanical misalignment [77]. That same year, they demonstrated resilience to geometric transformations including translation, scaling and rotation via physical data augmentation [78]. In 2024, Jia *et al*. established a training framework under the Gaussian–Schell model, maintaining classification accuracy exceeding 82% even under low-coherence illumination with a coherence length of 0.05 mm [79].

To break computational bottlenecks, researchers exploited parallelism further. Li *et al*. in 2023 used broadband sources to simulate the parallel execution of >180 independent matrix transformations and experimentally demonstrated dual-wavelength image permutation [80], with the WDM network shown in Fig. 3c. Fu *et al*. in 2025 proposed a dual-wavelength differential network utilizing complementary spatial frequency coverage, improving MNIST accuracy to 98.7%, an 11.4% increase over single-wavelength benchmarks [81].

In the polarization domain, Li *et al*. in 2022 achieved four independent parallel channels by embedding polarizer arrays in isotropic materials [82]. Chi *et al*. in 2025 extended this to terahertz metasurfaces using a single-layer birefringent design for dual-wavelength, dual-target recognition with 94.6% accuracy [83]. Additionally, Rahman *et al*. in 2024 demonstrated all-optical classification using orbital angular momentum modes, processing multiple superimposed mode inputs to achieve ∼85% accuracy on MNIST [84].

Addressing reconfigurability, Zhou *et al*. in 2021 constructed a diffractive processing unit (DPU) using spatial light modulators (SLMs) and digital micromirror devices, achieving a computing power exceeding 240.1 tera operations per second (TOPS) and 100% accuracy on the Weizmann video action recognition dataset [85]. The reconfigurable DPU is shown in Fig. 3d.

Recent efforts from 2024 to 2025 focused on integration and non-linearity. Liu *et al*. in 2025 proposed the shared optical core with lightweight transfer network architecture, where a compact 30 × 53 μm^2^ optical core achieved 95.9% average accuracy across three datasets via transfer learning [86]. Zhang *et al*. in 2025 utilized Ge microstructures to realize the first on-chip, low-loss all-optical non-linear activation, improving non-linear classification accuracy by 9.1% with a latency as low as 172 ps [87], as illustrated in the highly integrated all-optical non-linear D^2^NN (AON-D^2^NN) in Fig. 3e.

To overcome physical depth limits, Huang *et al*. in 2025 introduced a deep knowledge prior architecture. By parameterizing shallow metasurfaces with deep digital generative networks, they achieved omnidirectional 3D object recognition and complex distortion correction with a structural similarity index measure (SSIM) exceeding 0.95 in the microwave band [88].

D^2^NNs also excel in inverse problems and cross-domain applications. Luo *et al*. in 2022 validated D^2^NNs as inverse scattering solvers, imaging through random scatterers with fidelity exceeding 0.85 Pearson correlation coefficient [89]. Rahman *et al*. in 2021 demonstrated twin-image elimination in holographic reconstruction with an SSIM of ∼0.9 [90], while Mengu *et al*. in 2023 designed a snapshot multispectral imager achieving parallel imaging of 16 spectral channels via spatial routing [91].

In communications, Gao *et al*. in 2024 applied D^2^NNs to direction of arrival estimation, utilizing the super-oscillation effect to achieve 1° precision, surpassing the 4.37° of traditional methods [92]. The super-resolution D^2^NN architecture is shown in Fig. 3f. Mengu *et al*. in 2023 verified D^2^NNs as optical routers with error-free permutation of 25 channels [93]. Furthermore, recent research in 2025 showed that phase-encoded linear processors could parallelly approximate 35 non-linear functions with a mean absolute error of 0.06 [94].

### MZI-based PNNs

While D^2^NNs have evolved into a mature system utilizing free-space propagation for massive parallelism, challenges persist regarding limited non-linearity, manufacturing robustness and the trade-off between integration density and efficiency. To overcome these physical constraints and achieve compact, stable and reconfigurable computing, the field has pivoted toward PICs [7]. Leading this transition is the PNN architecture based on MZI arrays. Unlike static diffractive layers, this architecture utilizes guided-wave interference to flexibly control the amplitude and phase of light waves, enabling precise and programmable linear transformations within a chip-scale footprint. Its theoretical foundation can be traced back to 1994 when Reck *et al*. [95] proposed a universal optical implementation scheme for unitary matrices based on a recursive decomposition algorithm. In 2016, Clements *et al*. [96] introduced an optimized optical implementation scheme for unitary matrices based on Reck’s work. Compared to the Reck architecture, this optimized scheme effectively reduces optical depth, decreases light propagation losses and enhances robustness against manufacturing variations in optical components.

In 2017, Shen *et al*. [97] for the first time integrated a large-scale array comprising 56 programmable MZIs on an Si PIC, successfully demonstrating 4 × 4 optical matrix multiplication, as shown in Fig. 4a. Applying this system to a vowel recognition task, signifying the initial feasibility of large-scale integrated MZI arrays for constructing PNNs. However, limited by factors such as on-chip thermal crosstalk and the lack of an *in situ* training mechanism, the chip’s recognition accuracy (76.7%) remains lower than that of traditional 64-bit electronic computers (91.7%). In 2020, Shokraneh *et al*. [98] further optimized and proposed a diamond-shaped MZI cascade topology to address issues in the Reck architecture like sensitivity to phase errors and insertion loss caused by topological asymmetry and uneven optical path loss distribution. Simulations showed that the diamond mesh achieves 98.75% classification accuracy under ideal conditions and maintains ∼85% accuracy at 1 dB loss per MZI, compared to ∼75% for the Reck topology under identical conditions.

**Figure 4.**
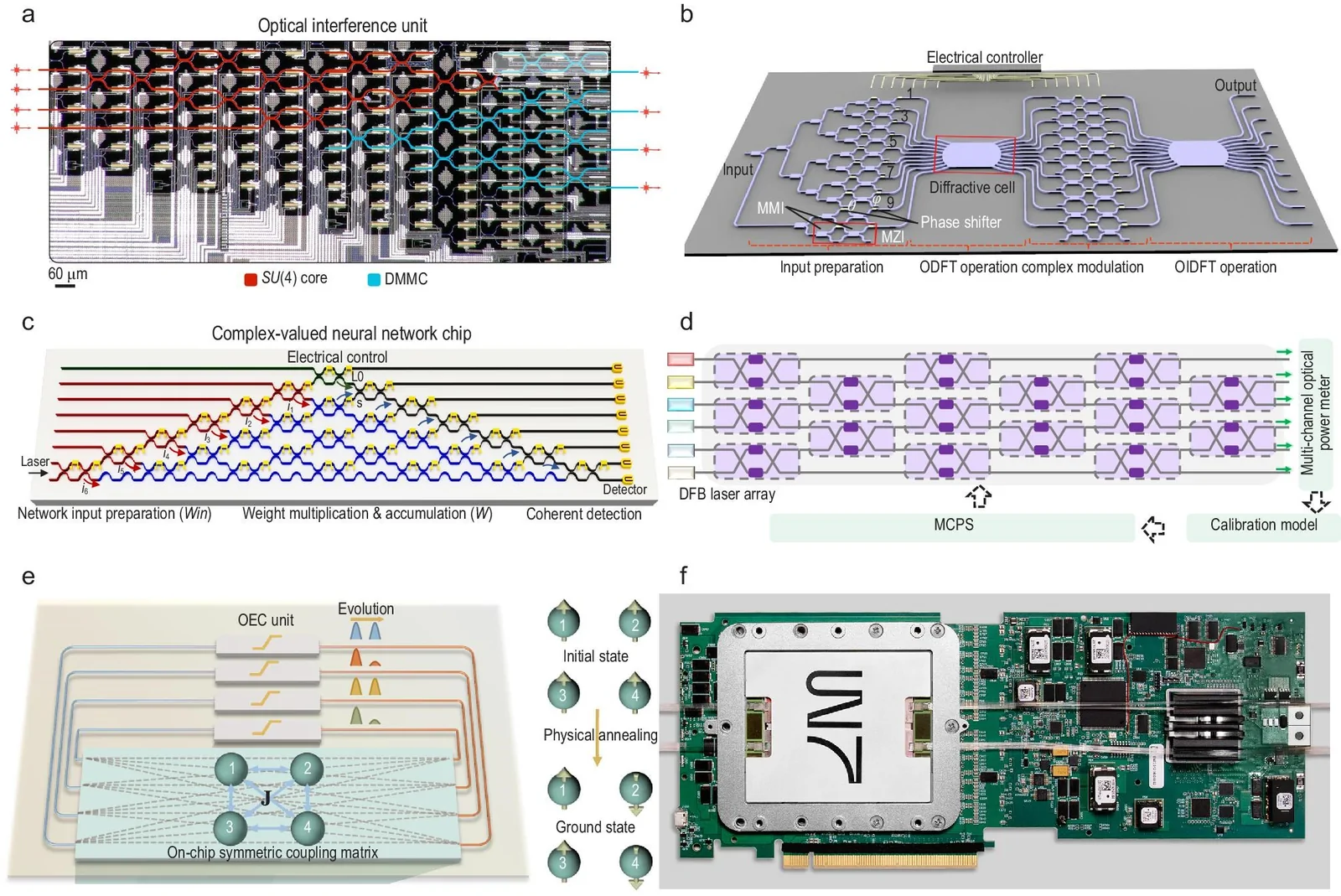
Architecture of MZI-based PNNs. (a) Programmable nanophotonic processor. Reproduced with permission from Ref. [97]. Copyright: 2017 Springer Nature. (b) Space-efficient integrated diffractive neural network (IDNN) architecture. Reproduced from Ref. [99] under a CC BY 4.0 license. (c) Complex-valued optical neural chip (ONC). Reproduced from Ref. [101] under a CC BY 4.0 license. (d) Calibration system based on DRC. Reproduced from Ref. [106] under a CC BY 4.0 license. (e) Monolithically integrated optical Ising machine. Reproduced from Ref. [107] under a CC BY-NC-ND 4.0 license. (f) Universal photonic AI processor. Reproduced with permission from Ref. [108]. Copyright: 2025 Springer Nature.

Subsequently, MZI-based PNNs have evolved mainly along three paths: scaling and efficiency optimization, aiming to overcome bottlenecks like rapidly increasing component count and chip area through simplified designs; expanding complex computing capabilities, extending MZI arrays from real-valued to complex‑number and logical operations; and enhancing noise and fault tolerance. In the area of scaling and efficiency optimization, in 2022, Zhu *et al*. [99] proposed an integrated diffractive PNN architecture based on MZIs, as shown in Fig. 4b. This architecture requires only O(*N*) MZIs to perform photonic computing tasks like parallel Fourier transforms and convolutions, achieving a hardware footprint of only 0.53 mm^2^ and a power consumption of 17.5 mW, approximately a 10-fold reduction compared to conventional MZI-based designs, while attaining experimental classification accuracies of 98.3% on Iris and 89.3% on MNIST. In 2023, Wu *et al*. [100] reported a novel simplified MZI network structure for real-valued optical matrix computation. This structure requires only *N*^2^ phase shifters and has an optical depth of *N* + 1, achieving a mean matrix correlation of 0.99 984 for 4 × 4 configurations and demonstrating Iris classification with 98.1% training accuracy.

In the area of the expansion of complex computing capabilities, in 2021, Zhang *et al*. [101] presented a method for implementing complex-valued neural networks on a PIC, as shown in Fig. 4c. This complex-valued network achieved nearly 100% accuracy in classifying non-linear datasets. In 2025, Xie *et al*. [102] reported a programmable complex-valued matrix-vector computation chip, realizing high-speed complex-valued MVM at a computing speed of 1.28 TOPS with a 16-channel processor operating at 40 Gbaud, and demonstrated 97% accuracy on handwritten digit recognition. In addition, Zhang *et al*. [103] introduced a novel architecture for photonic logical tensor computation and constructed the first photonic general-purpose logical tensor core. Its total computational capacity exceeded 1 Tbit/s.

In terms of noise suppression and fault tolerance enhancement, in 2019, Fang *et al*. [104] proposed an optimization strategy by analyzing performance across MZI array topologies, showing that the GridNet architecture achieves ∼98% MNIST accuracy under ideal conditions while the FFTNet architecture, though lower in peak accuracy (∼95%), exhibits significantly greater robustness under component imprecisions, thereby revealing the trade-off between network robustness and computational expressiveness. In 2024, Shafiee *et al*. [105] developed a device-to-system modeling framework capable of evaluating the cumulative impact of loss and crosstalk noise in different MZI topologies on network performance, quantifying that for a 16-input, two-hidden-layer Clements SP-NN, the accumulated optical loss can reach 38 dB with an SNR as low as 7.3 dB, leading to an inference accuracy degradation of up to 84.6% on the MNIST task. In 2025, Yan *et al*. [106] tackled the computational errors in multiwavelength optical information processing systems caused by frequency-selective responses by proposing a calibration method based on deep reinforcement learning (DRC), as shown in Fig. 4d. The method learns optimal calibration strategy through real-time system interaction and adapts to diverse environments, including MZI arrays, achieving convergence within 21 iterations and reducing output errors by 78.0% in DCF-based systems compared to uncalibrated outputs, and by 85.4% in MZI array-based systems compared to the standard voltage configuration.

In 2025, research on MZI-based photonic computing architectures achieved critical breakthroughs in system-level applications and overall performance. Wu *et al*. [107] proposed a monolithically integrated optical Ising machine based on MZI and optoelectronic coupled oscillators, featuring an ultra-compact OEC unit footprint of 0.01 mm^2^ and a power efficiency of 4 mW per unit, with a reconfigurable coupling matrix achieving a mean fidelity of 0.986 and a spin evolution time of 150 ns, demonstrating significant improvements in three key metrics: integration density, energy efficiency and convergence speed, as shown in Fig. 4e. Ahmed *et al*. [108] developed a photonic processor capable of executing complex AI models, integrating four 128 × 128 photonic tensor cores to deliver 65.5 trillion ABFP16 operations per second at 78 W of electrical power and 1.6 W of optical power, and successfully running ResNet-18 (an 18-layer deep residual convolutional network), BERT-Tiny (a compact variant of the bidirectional encoder representations from transformers language model) and Atari DRC models with near-electronic precision, as shown in Fig. 4f. By employing a hybrid architecture design, this work deeply integrates the advantages of high-speed linear operations in photonics with the flexible logic control capabilities of electronics.

### WDM-based PNNs

Overall, the core advantages of MZI-based PNNs stem from their interference-based mechanism, offering unique benefits for efficient linear operations. However, practical scaling faces multifaceted difficulties. High-density integration introduces thermal crosstalk and phase instability, while fabrication imperfections necessitate complex calibration mechanisms to maintain accuracy. Most critically, the chip footprint increases significantly with matrix scale, creating a spatial bottleneck that limits monolithic integration. To transcend these physical area constraints, researchers have expanded into the spectral domain. PNNs based on WDM utilize distinct wavelengths as independent channels to achieve high-throughput data processing via spectral parallelism. Over the past decade, this field has evolved from early theoretical validation to integrated microcomb-driven architecture centered on time-space-wavelength multidimensional multiplexing.

Early exploration focused on mechanism validation and capacity analysis. The Princeton group in 2014 proposed the ‘Broadcast-and-Weight’ protocol, defining an architecture that uses wavelength as neuron identity, weights signals via MRRs and performs fan-in summation through incoherent superposition at the detector, thereby resolving electronic interconnect bandwidth bottlenecks [109]. Subsequently, Tait *et al*. in 2015 built a physical prototype demonstrating WDM weighted addition for principal component analysis of 1 Gbps signals [110]. Addressing physical limitations, Tait *et al*. in 2016 analyzed crosstalk in dense WDM and introduced a cross-weight power penalty to prove the theoretical feasibility of scaling to hundreds of channels, surpassing previous conservative estimates of ∼30 channels [111]. The Broadcast-and-Weight protocol is illustrated in Fig. 5a. To enhance robustness, the two-pole design proposed in 2018 significantly improved channel density and stability [112].

**Figure 5.**
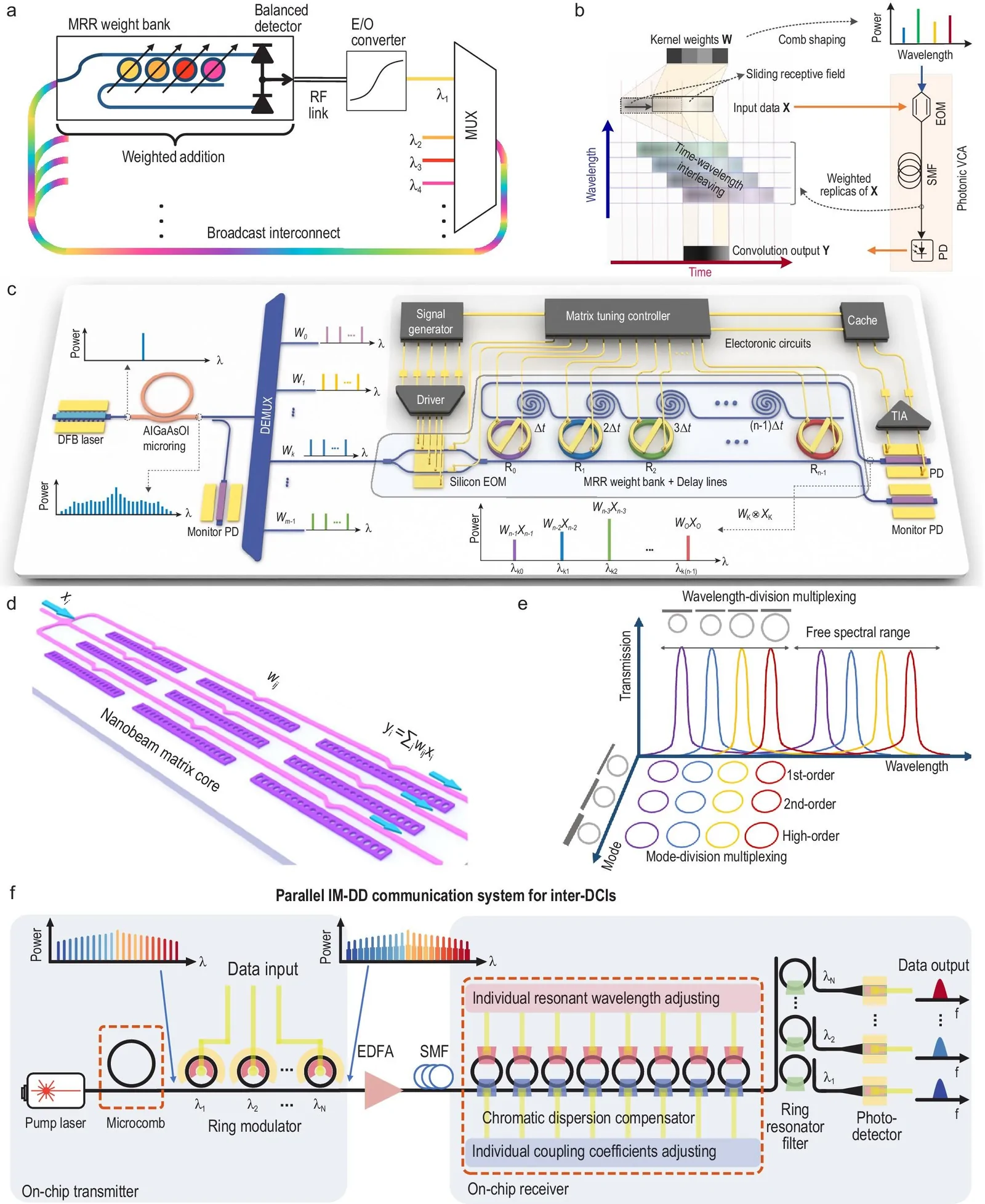
Architecture of WDM-based PNNs. (a) Broadcast-and-weight protocol. Reproduced with permission from Ref. [111]. Copyright: 2016 IEEE. (b) Photonic vector convolutional accelerator. Reproduced with permission from Ref. [113]. Copyright: 2021 Springer Nature. (c) Integrated photonic processing unit. Reproduced from Ref. [114] under a CC BY 4.0 license. (d) PCNC matrix core. Reproduced from Ref. [117] under the Optica Open Access Publishing Agreement. (e) Multidimensional photonic computing. Reproduced with permission from Ref. [121]. Copyright: 2025 Optica Publishing Group. (f) The parallel transmission system including the chromatic dispersion compensator. Reproduced from Ref. [126] under a CC BY 4.0 license.

The maturation of microcomb enabled order-of-magnitude improvements in computing power [31]. Xu *et al*. in 2021 achieved a milestone using a soliton crystal microcomb to generate 90 wavelengths combined with dispersive fiber-based time-wavelength interleaving. This system realized an ultra-high convolutional computing power of 11 TOPS and successfully demonstrated the processing of large-scale images with 250 000 pixels [113], using the photonic vector convolutional accelerator shown in Fig. 5b. Subsequent research focused on integration and complex processing. Bai *et al*. in 2023 integrated dark pulse microcomb sources with computing units on an AlGaAsOI platform, achieving a density of 1.04 TOPS/mm^2^ and 9-bit weight control precision, eliminating reliance on bulky desktop light sources [114]. The integrated photonic processing unit is shown in Fig. 5c. For phase-dependent scenarios, Bai *et al*. in 2025 proposed a complex-domain convolutional accelerator. By using synthetic wavelengths to carry real and imaginary parts, they achieved a complex inference capability exceeding 2 TOPS and an accuracy of 83.8% in synthetic aperture radar image recognition [115].

To overcome MRR FSR limits, Zhang *et al*. in 2023 utilized four-wave mixing to generate high-dimensional bases and linear MRR arrays to implement arbitrary Boolean logic at 10 Gbit/s [116]. In 2024, the group replaced MRRs with photonic crystal nanobeam cavities (PCNCs), reducing device size to 20 × 0.5 μm while eliminating FSR constraints and improving thermal tuning efficiency by three times [117], based on the PCNC matrix core shown in Fig. 5d. That same year, they utilized the wavelength routing of arrayed waveguide gratings to physically simulate convolution kernel sliding, achieving redundancy-free computing in a single clock cycle with a theoretical density of 8.53 TOPS/mm^2^ [118].

Expanding beyond single-wavelength dimensions, Yin *et al*. in 2023 pioneered the hybrid integration of mode-division multiplexing (MDM) and WDM on an Si chip, employing a three-mode demultiplexer/multiplexer alongside MRR arrays to perform MVM; combined with a transformation mapping method that enables real-number-field computation without balanced PDs, the fabricated device achieved image edge-detection convolution and letter pattern recognition at 22.38 Gbaud with a computing density of 1.37 TOPS/mm^2^ and 4.48 fJ per operation energy efficiency [119]. Latifpour *et al*. in 2024 constructed a hyperspectral in-memory computing architecture using OFCs and SLMs, demonstrating a throughput of 2.048 TOPS [120]. Jia *et al*. in 2025 introduced MDM, utilizing TE0 and TE1 modes within a single waveguide to create a 2D wavelength-mode computing space [121], enabling the multidimensional photonic computing architecture shown in Fig. 5e. Concurrently, Meng *et al*. in 2025 demonstrated a high-computing-density optical tensor processing unit based on a single passive MRR, where kernel weights were assigned by tuning laser wavelengths and input dimensionality was expanded through hybrid lightwave–microwave multiplexing across wavelength, time and microwave-frequency domains, achieving an experimental computing density of 34.04 TOPS/mm^2^ and 96.41% accuracy on MNIST handwritten digit recognition at 30.67 Gbaud [122]. Bi *et al*. developed digital subcarrier multiplexing in 2025, superimposing electrical subcarriers on optical wavelengths to achieve a throughput of 128 GOPS with a dual-wavelength configuration [123]. Prapas *et al*. in 2025 combined chiplet technology, a modular photonic integration approach in which separately fabricated functional dies (chiplets), each optimized for a specific function (e.g. modulation, routing and detection), are interconnected to form a complete photonic system, with SiGe EAM arrays to build a time–space–wavelength multiplexed photonic tensor core, achieving an aggregate power of 2.56 TOPS [124]. The same group also demonstrated a hyperdimensional architecture based on a coherent crossbar and WDM with a monolithic power of 0.96 TOPS [125].

WDM computing has expanded to communication, sensing and scientific tasks. Liu *et al*. in 2024 used WDM MRMs for dispersion compensation, optimizing energy efficiency to ∼0.3 pJ/bit [126]. The parallel transmission system including the chromatic dispersion compensator is shown in Fig. 5f. The group also utilized WDM and dispersion delay for optical forward error correction encoding, reducing latency to ∼2.1 ns [127]. Yu *et al*. in 2025 developed a time-wavelength multiplexing (TWM) accelerator for distributed acoustic sensing, achieving 90.8% event recognition accuracy at 10 Gbaud [128]. Addressing precision, a HUST group in 2024 demonstrated a 32-bit digital multiplier based on time–space multiplexing [129]. In 2025, HUST researchers showcased the potential of TFLN platforms for ray tracing, utilizing 1-bit analog-to-digital converter (ADC) technology and WDM parallelism to support real-time physical rendering [130].

In parallel with the advancement of volatile MRR-based WDM architectures, the incorporation of non-volatile PCM weight banks has opened an orthogonal path toward zero-static-power photonic tensor cores. In 2021, Feldmann *et al*. demonstrated an integrated photonic tensor core that combines an Si_3_N_4_ soliton microcomb with GST phase-change memory arrays on a waveguide crossbar, performing parallel MVM via WDM [131]. This architecture achieved tera-MAC-per-second throughput at over 14 GHz bandwidth with 5-bit precision, and was validated by executing convolutional image processing and MNIST digit recognition with 95.3% accuracy. Since the PCM weight elements retain their programmed states without continuous power, this approach fundamentally eliminates the static thermal tuning overhead associated with volatile MRR-based weight banks.

Expanding the parallelism of PCM-based architectures beyond the spatial and wavelength domains, in 2023, Dong *et al*. introduced RF modulation as a third degree of freedom [132]. By multiplexing 50 RF components across two WDM channels on an electro-optically controlled photonic tensor core with GST memory elements, the system achieved a parallelism of 100, two orders of magnitude above prior photonic crossbar implementations, and was configured for edge cloud computing to synchronously convolve 100 clinical electrocardiogram signals for cardiac risk assessment with 93.5% accuracy. In 2024, the same group demonstrated that partially coherent light can further enhance parallelism by eliminating phase-fluctuation-induced intensity noise, allowing the same optical band to be shared across all input channels and yielding an *N*-fold parallelism gain over coherent-light schemes [133]. This principle was validated on both a 3 × 3 PCM photonic memory tensor core that classified Parkinson’s disease patient gaits with 92.2% accuracy, and a 9 × 3 Si photonic tensor core with integrated EAMs achieving 0.108 TOPS and 92.4% accuracy on MNIST digit classification.

### CMod-based PNNs

In addition to resonator-based WDM architectures, simplified coherent schemes utilizing CMod have emerged as a powerful alternative. Unlike resonance-sensitive designs, the CMod-based PNN architecture achieves dynamic signal control and parallel processing by simply connecting multiple phase or intensity modulators in series. By leveraging high-speed EO modulation, this approach enables efficient MVM with significantly reduced structural complexity, offering a robust pathway for high-bandwidth optical processing. In 2022, Sludds *et al*. [134] proposed Netcast, a photonic edge computing architecture that integrates CMod with WDM technology. In this architecture, modulators at the edge node load the data to be inferred. After transmission through optical fiber to the cloud server, modulators in the server load the weights. This approach achieved ultra-low-energy matrix computation at 40 aJ/multiplication operation and an accuracy of 98.8% on the MNIST handwritten digit recognition task.

To further construct a complete deep neural network based on a CMod architecture, in 2023, Zhong *et al*. [135] presented Lightning, a reconfigurable optoelectronic smart network interface card. It utilizes digital circuits to schedule the data flow, combining WDM and CMod to perform matrix operations, while non-linear activation is executed by the digital circuits. This successfully constructs a complete deep neural network at the hardware level. Experiments demonstrated that the system supports photonic MAC operations with an accuracy of 99.25% and achieves an end-to-end inference rate of 4.055 GHz. To push the performance boundaries, the research focus has shifted toward TFLN material systems, which offer stronger EO effects and lower optical losses. In 2024, Lin *et al*. [136] reported an integrated photonic tensor core based on TFLN platform, with flexible configuration of input and output dimensions, a computing speed of 120 GOPS and a weight update rate of 60 GHz. This architecture introduced a dual-wavelength injection scheme for the first time, enabling the multiplication of two negative numbers in a PNN, which allows it to handle unsupervised learning tasks. The integrated photonic tensor core on TFLN is shown in Fig. 6a. In 2025, Hu *et al*. [137] further advanced this architecture based on the TFLN platform. By leveraging TFLN modulators with bandwidth exceeding 40 GHz and ultra-high modulation efficiency, they achieved a computing speed of 43.8 GOPS per channel and an energy consumption level of 0.0576 pJ/operation, significantly outperforming conventional EO conversion schemes. The image of one integrated TFLN computing core with the building blocks is shown in Fig. 6b.

**Figure 6.**
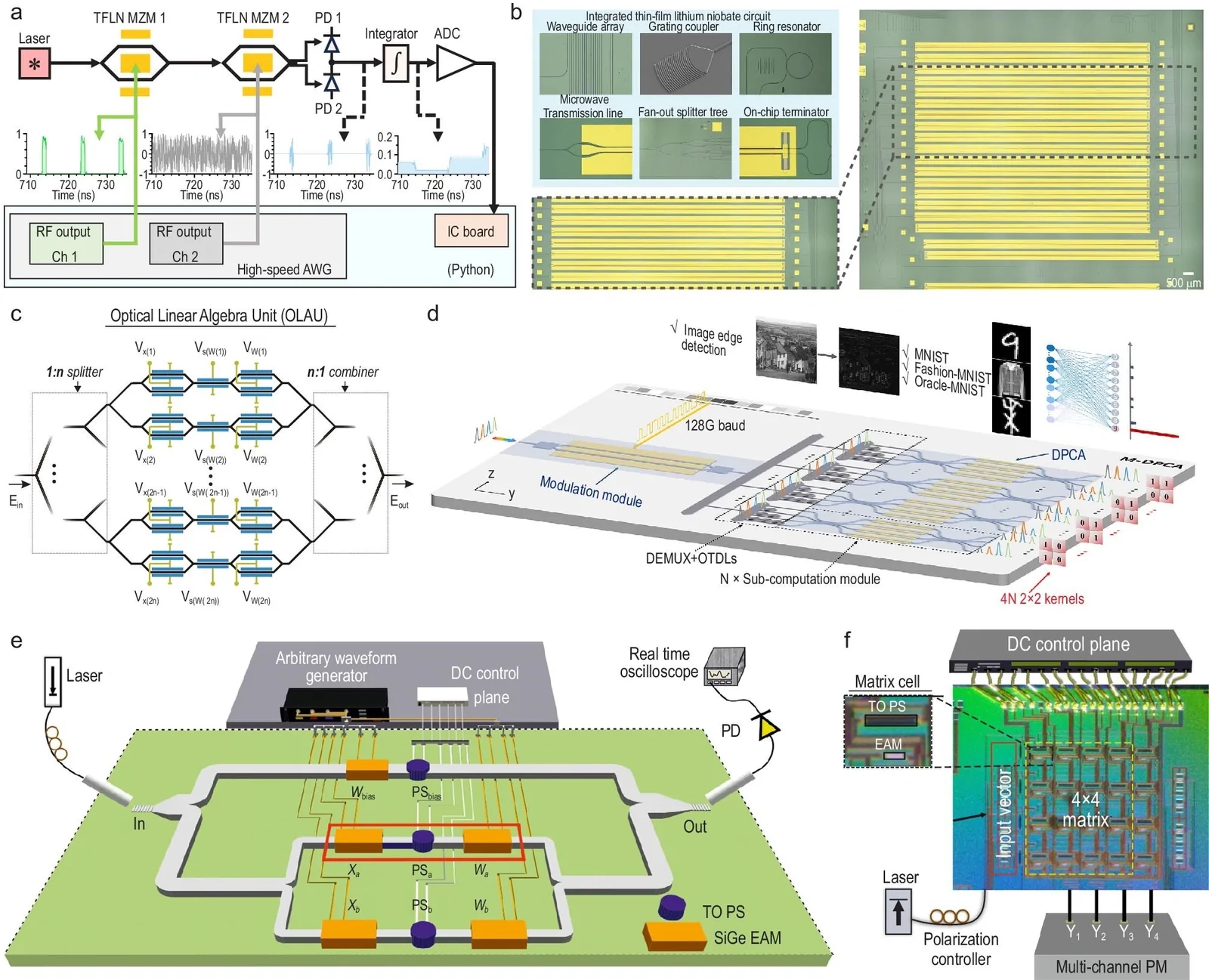
Architecture of CMod-based PNNs. (a) Integrated photonic tensor core on TFLN. Reproduced from Ref. [136] under a CC BY 4.0 license. (b) One integrated TFLN computing core with the building blocks. Reproduced from Ref. [137] under a CC BY-NC-ND 4.0 license. (c) The *n*-input OLAU. Reproduced with permission from Ref. [138]. Copyright: 2020 IEEE. (d) Task-driven integrated DPCA on TFLN. Reproduced with permission from Ref. [140]. Copyright: 2025 Wiley-VCH GmbH. (e) SiGe EAM-based COLN layout. Reproduced from Ref. [141] under a CC BY 4.0 license. (f) High-fidelity 4 × 4 coherent Si photonic Xbar chip. Reproduced from Ref. [142] under a CC BY 4.0 license.

In addition to the fundamental structure of directly cascaded intensity modulators, the generalized CMod-based PNN architecture also includes configurations where modulators are interconnected via other functional components. For instance, in 2019, Mourgias-Alexandris *et al*. [138] proposed a coherent linear photonic neuron based on an extended in-phase and quadrature (IQ) modulator. This architecture incorporates an additional MZM into each branch of a conventional IQ modulator and introduces a phase modulator to independently represent positive and negative weights, thereby forming an extended IQ modulator capable of simultaneously modulating the amplitude and phase of the optical signal. The *n*-input optical linear algebra unit (OLAU) is shown in Fig. 6c. However, a single neuron cannot support complex tasks. Therefore, the research focus of CMod-based PNNs has shifted from realizing the functions of individual neurons to building systems that support large-scale matrix operations. In 2024, Kovaios *et al*. [139] reported a universal matrix multiplier based on an Si photonic crossbar. This design employs a hybrid time-domain and space-domain multiplexing technique, enabling the execution of matrix operations that exceed the physical size of the hardware, utilizing the time–space multiplexed layout. In 2025, Zhang *et al*. [140], leveraging the TFLN platform, proposed a distributed parallel computing array composed of multiport directional couplers and TFLN modulators. In this array, each submodule can independently execute a 2 × 2 convolution kernel operation. The task-driven integrated distributed parallel computing architecture (DPCA) is illustrated in Fig. 6d.

For photonic computing architectures based on CMod, achieving a high-performance architecture has always been a key challenge on the path to practical implementation. In 2023, Giamougiannis *et al*. [141] employed an Si-based coherent linear neuron (COLN) architecture, combining high-speed SiGe EAMs to load the input and weights separately, to achieve the first demonstration of optical block matrix multiplication at a rate of 50 GHz, using the SiGe EAM-based COLN layout shown in Fig. 6e. In addition to pursuing high-speed computing, ensuring the reliability and accuracy of computational results is also a key issue in high-performance CMod-based PNN architectures. In 2024, Moralis-Pegios *et al*. [142] similarly used SiGe EAMs for input and weight encoding. They utilized thermo-optic phase shifters for fine-tuning the phase and employed a hardware-aware programming model to improve system calibration accuracy. In experiments performing 10 000 arbitrary linear transformations, this architecture achieved a fidelity of 99.997% ± 0.002%, setting a record at the time. The high-fidelity 4 × 4 coherent Si photonic Xbar chip is shown in Fig. 6f. In 2025, Xu *et al*. [143] introduced an integrated thermo-optic modulator based on a PCNC and an indium tin oxide (ITO) microheater. They reported the first demonstration of a PNN supporting 5-bit quantization, achieving a mean quantization error of only 0.0107.

Having examined the four mainstream photonic computing core architectures individually (D^2^NN, MZI-based, WDM-based and CMod-based PNNs), it becomes evident that each paradigm leverages fundamentally different physical mechanisms, resulting in distinct trade-off profiles across key performance metrics. To facilitate a systematic and side-by-side evaluation, Table 1 summarizes the comparative standing of these four architectures across seven critical dimensions: integration density, scalability, energy efficiency, operational stability, reconfigurability, computing precision and throughput. This multidimensional comparison aims to provide readers with a concise reference for identifying the most suitable architecture for specific application scenarios, while also highlighting the inherent design trade-offs that currently prevent any single approach from simultaneously excelling across all metrics.

**Table 1.** Comparison of computing architectures across key performance dimensions.

Dimension	D^2^NN	MZI-based	WDM-based	CMod-based
Integration density	Low (free-space optics; improving with on-chip architectures)	Medium (hundreds-of-micrometer-scale MZI arms limit footprint)	High (micrometer-scale MRR footprint)	Medium (mm-scale modulator arms limit integration density)
Scalability	Low (single-layer neuron count scales favorably; on-chip integration constrained by limited input waveguide dimensions and insertion loss with depth)	Medium (O(*N*^2^) components for *N* × *N* matrix; cumulative insertion loss limits scale)	High (scales with wavelength channels; 10^1^–10^2^ channels demonstrated)	Medium (scales with modulator cascade depth; noise accumulation is the key limiting factor)
Energy efficiency	High (passive diffractive propagation; near-zero dynamic power consumption)	Low (active thermo-optic phase tuning consumes ∼10–20 mW per shifter; scale with O(*N*^2^) components for *N* × *N* matrix)	Low (MRR thermal tuning overhead)	Medium (modulator amplifier power consumption dominates overall energy budget)
Operational stability	High (passive operation eliminates active drift; fabrication tolerance and alignment precision affect initial accuracy but not runtime stability)	Low (each MZI arm requires a thermo-optic phase shifter; large-scale arrays suffer from interheater thermal crosstalk)	Low (MRR resonance highly sensitive to thermal and fabrication variation)	Medium (non-resonant structure; less sensitive than MRR but subject to modulator bias drift)
Reconfigurability	Low (fixed once fabricated; SLM-based free-space systems are reconfigurable)	High (electrically tunable phase shifters enable arbitrary unitary matrices)	High (thermally or electrically tunable MRRs)	High (voltage-controlled modulators with fast response)
Computing precision	Low (free-space implementations limited by 3D-printing resolution; on-chip integration fabricated via lithography or electron beam lithography achieve higher precision)	Medium (single MZI achievable high resolution; cumulative phase error and thermal crosstalk degrade effective precision in large-scale meshes)	High (continuous analog tunability provides broad weight resolution; thermal stabilization and wavelength locking techniques further enhance effective precision)	Medium (precision primarily determined by Digital-to-Analog Converter resolution and modulator linearity; TFLN modulator offers good transfer linearity)
Throughput	High (light-speed single-pass inference with massive spatial parallelism; bottlenecked by front-end input encoding rate)	Low (input modulation bandwidth reaches GHz range; O(*N*^2^) component scaling constrains matrix size and parallelism)	High (wavelength parallelism linearly scales throughput with channel count)	High (GHz-bandwidth modulators enable high-speed per-channel inference)

The D^2^NN architecture derives its core advantages from a passive diffractive propagation mechanism, where inference is completed through a single light-speed pass without any active tuning. This passive nature simultaneously endows the architecture with near-zero dynamic power consumption and excellent operational stability free from runtime drift, making D^2^NNs among the most energy-efficient computing paradigms. The massive spatial parallelism inherent in free-space implementations further translates this efficiency into high throughput for fixed tasks. However, the very passivity that grants these benefits also imposes a fundamental trade-off: once the diffractive layers are fabricated, their physical parameters are permanently fixed, severely limiting reconfigurability and restricting each device to its originally trained task. Computing precision is likewise tied to the fabrication process—free-space versions relying on 3D printing offer relatively coarse feature control, while emerging on-chip implementations fabricated via deep ultraviolet (DUV) lithography or electron beam lithography (EBL) achieve notably improved weight accuracy. Moving toward on-chip integration alleviates the bulky footprint of free-space systems, yet scalability remains low, as input waveguide dimensions and cumulative insertion loss with increasing network depth continue to constrain the achievable scale. Overall, D^2^NNs are best suited for inference-only edge applications with fixed tasks, where their passive operation and extreme energy efficiency outweigh the lack of runtime reconfigurability.

In contrast to the passive and fixed nature of D^2^NNs, MZI-based PNNs adopt a fundamentally different design philosophy that prioritizes runtime programmability through active guided-wave interference. MZI-based PNNs derive their core strength from electrically tunable phase shifters capable of realizing arbitrary unitary matrix transformations, providing the highest degree of reconfigurability among the four architectures. This full programmability enables versatile deployment across diverse neural network models without hardware redesign, and the precise phase control inherent in the interference-based mechanism supports medium-to-high computing precision at the single-device level. Yet this flexibility comes at a considerable physical cost. Implementing an *N* × *N* matrix requires O(*N*^2^) MZI components, which not only limits scalability due to cumulative insertion loss with increasing depth, but also drives up energy consumption, as each MZI arm demands an active thermo-optic phase shifter whose aggregate power grows rapidly with array size. The dense packing of heaters further compounds the problem by introducing interheater thermal crosstalk, which degrades phase accuracy and undermines operational stability in large-scale arrays. Integration density is additionally constrained by the relatively large footprint of the interferometric arms, while throughput, though benefiting from GHz-range modulation bandwidth, is ultimately capped by these spatial scaling limitations. Consequently, MZI-based PNNs are most appropriate for applications that demand full programmability and model flexibility, particularly where frequent weight reconfiguration is required.

While MZI-based PNNs achieve reconfigurability in the spatial domain through cascaded interferometers, WDM-based PNN architectures exploit an orthogonal design strategy—encoding computations across the spectral domain to pursue higher throughput through wavelength parallelism. The WDM-based architecture maximizes computational throughput by exploiting this parallelism, where each wavelength channel independently carries a separate computation. Since throughput scales linearly with the number of available channels, this approach offers the most favorable scaling pathway among the four architectures, while the microscale MRR footprint simultaneously enables high integration density. The continuous analog tunability of MRR transmission further provides high weight resolution, which can be enhanced by mature thermal stabilization and wavelength locking techniques from the Si photonics ecosystem. However, the resonant mechanism that enables such compact and precise weighting also introduces the architecture’s primary vulnerability: MRR resonance wavelengths are highly sensitive to thermal fluctuations and fabrication variations, resulting in low operational stability without active feedback control. The thermal tuning and locking loops required to maintain stable operation in turn introduce additional power overhead, placing overall energy efficiency at a medium level. As the system scales to denser channel configurations, interchannel crosstalk becomes an increasing concern that can further degrade system-level performance. WDM-based PNNs are therefore ideally suited for high-throughput convolutional and MVM workloads demanding massive parallelism, particularly in controlled environments where active stabilization can be reliably maintained.

Complementary to the resonance-based weighting mechanism of WDM architectures, CMod-based PNNs adopt a non-resonant approach that leverages direct EO modulation, offering a distinct trade-off profile in stability and architectural simplicity. CMod-based PNNs offer a pragmatic balance between performance and architectural simplicity by cascading high-speed EOMs for dynamic signal control. A defining advantage of this approach is the non-resonant modulator structure, which is substantially less sensitive to thermal and fabrication variations than MRR-based designs, yielding notably better operational stability. This robustness extends to computing precision, which is governed primarily by DAC resolution and modulator transfer linearity, both engineerable parameters, rather than by intrinsic physical constraints, with MZM and particularly TFLN platforms offering excellent linearity and considerable room for further improvement. The high modulation bandwidth of these devices directly enables high per-channel throughput, while voltage-controlled tunability with fast response times ensures strong reconfigurability. Nevertheless, the architecture faces its own scaling bottleneck: noise accumulation along the modulator cascade limits the achievable network depth, and the relatively large modulator arm lengths constrain integration density. Moreover, the modulator amplifier circuitry dominates the overall energy budget, placing energy efficiency at a medium level. CMod-based PNNs are thus well-positioned for high-speed inference acceleration at moderate scale, where their combination of stability, speed and reconfigurability provides a robust and practically deployable solution.

The four architectures discussed above, D^2^NN, MZI-based, WDM-based and CMod-based PNNs, primarily exploit the linear properties of light propagation, interference and modulation to implement high-speed MVM and convolution operations. However, linear transformations alone are insufficient for constructing a functionally complete neural network. Without non-linear activation between successive layers, a multilayer network degenerates into a single linear mapping, fundamentally limiting its representational capacity and preventing it from learning complex decision boundaries. Therefore, the non-linear activation function is an indispensable functional component shared by all four architectures, regardless of their specific physical implementation. Given its cross-architecture universality, we present the non-linear activation function as a dedicated subsection below to provide a unified discussion of the current physical implementation approaches and the key challenges that remain in achieving efficient, integrable optical non-linearity for deep PNNs.

### Non-linear activation functions in PNNs

The PNN architectures reviewed thus far excel at performing linear matrix multiplications. However, the realization of deep feature extraction and complex decision-making necessitates the integration of non-linear activation functions. Given the physical challenges associated with efficient optical non-linearity, conventional implementations typically offload these operations to external digital electronics. Yet, this reliance on digital post-processing imposes severe latency and power consumption penalties, thereby negating the inherent speed advantages of photonics. To circumvent these bottlenecks, recent research has pivoted toward developing dedicated hardware implementations for *in situ* activation. These emerging solutions can be broadly classified into two distinct paradigms: EO hybrid schemes and all-optical schemes.

The EO hybrid scheme primarily implements non-linear activation based on photodetection and EO modulation, offering advantages such as ease of implementation and integration. However, its response speed and energy efficiency are limited by the EO conversion process. Currently, the mainstream on-chip implementation of the EO hybrid scheme is the detection-modulation approach: the optical signal output from the previous network layer is first converted into an electrical signal by a PD, then amplified to drive an EOM with a non-linear transfer function, thereby achieving non-linear transformation in the optical domain. In 2019, Mourgias-Alexandris *et al*. [144] realized a non-linear optical neuron based on the Sigmoid function for the first time using a differentially biased semiconductor optical amplifier Mach–Zehnder interferometer (SOA-MZI). This architecture successfully constructed a tunable non-linear response by cascading a deeply saturated SOA-MZI with another SOA stage. In 2021, Amin *et al*. [145] first proposed an integrated absorption modulator based on an ITO–graphene heterojunction, which achieved an optical transfer function similar to leaky rectified linear unit (ReLU) by precisely controlling the light absorption intensity of the ITO film via voltage, with a modulation efficiency of 0.132 dB/μm, a switching energy of 2.43 pJ and an insertion loss below 2 dB, using the ITO–graphene-heterojunction-integrated absorption modulator shown in Fig. 7a. To further extend the operational optical domain, in 2024, Zhang *et al*. [146] constructed a transparent optoelectronic neuron array through the heterogeneous integration of two-dimensional transparent phototransistors with liquid crystal modulators, realizing non-linear self-amplitude modulation for broadband incoherent light for the first time, with a device-modeling-predicted ultra-low non-linear contrast threshold intensity of 56 μW/cm² and an energy consumption of only 69 fJ per photonic activation after device optimization. The transparent optoelectronic neuron array is shown in Fig. 7b. In the same year, to fully leverage the advantages of high computational speed and low power consumption offered by system-level PNNs, Bandyopadhyay *et al*. [147] reported a fully integrated coherent PNN supporting end-to-end inference and *in situ* training, named FICONN. This architecture utilizes photocurrent to directly drive MRMs, achieving programmable non-linear activation functions and constructing a complete system-level PNN, with an end-to-end latency of 410 ps and a vowel classification accuracy of 92.5% using 132 on-chip tunable parameters.

**Figure 7.**
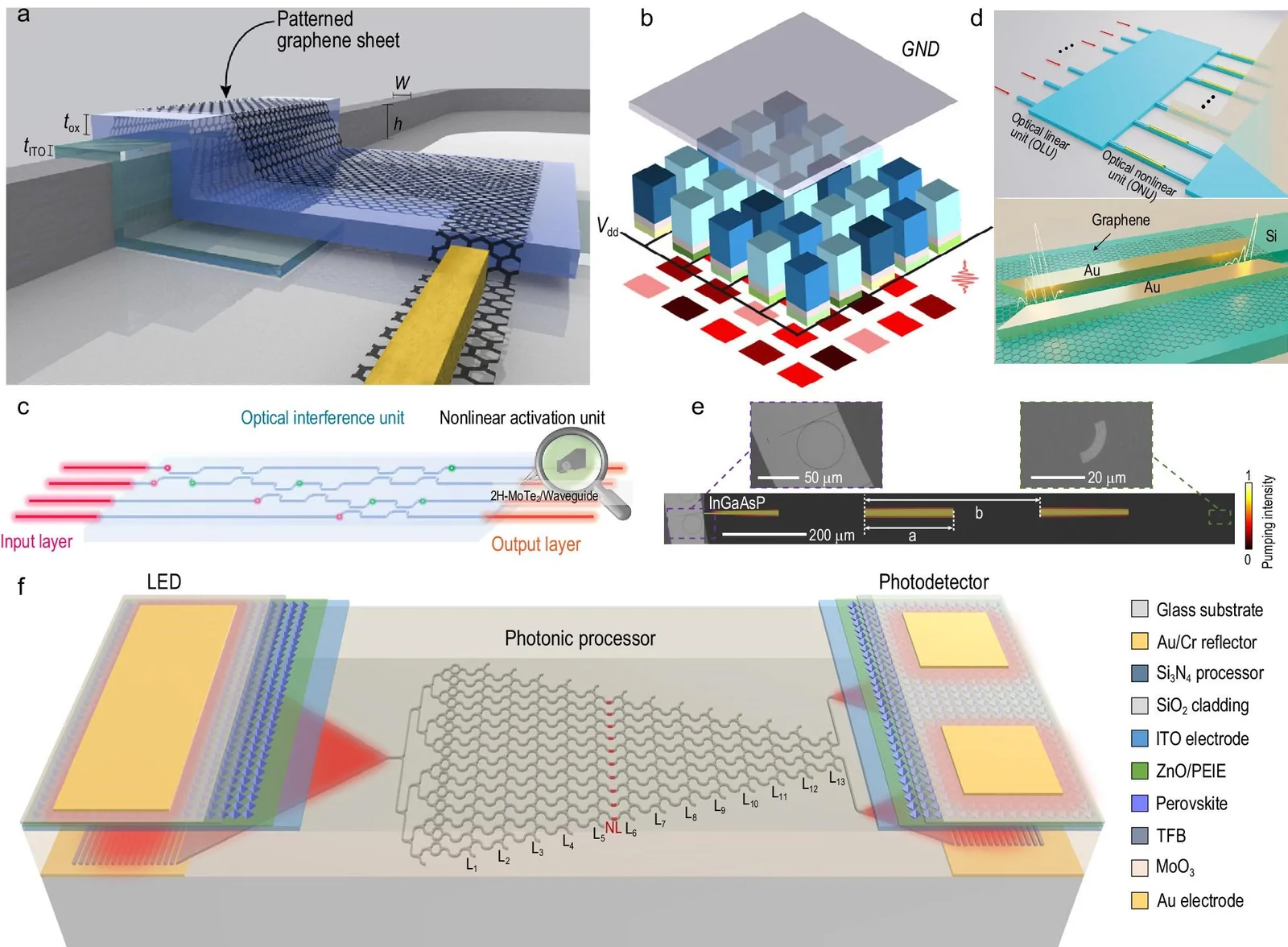
Architecture of non-linear activation functions in PNNs. (a) ITO–graphene heterojunction integrated absorption modulator. Reproduced from Ref. [145] under a CC BY 4.0 license. (b) Transparent optoelectronic neuron array. Reproduced from Ref. [146] under a CC BY 4.0 license; (c) MoTe_2_-based ultra-broadband all-optical activation unit. Reproduced from Ref. [148] under a CC BY-NC-ND 4.0 license. (d) Ultra-fast Si/graphene optical non-linear activator. Reproduced with permission from Ref. [149]. Copyright: 2024 Wiley-VCH GmbH. (e) Field-programmable photonic non-linearity architecture. Reproduced with permission from Ref. [151]. Copyright: 2025 Springer Nature. (f) Hetero-integrated perovskite/Si_3_N_4_ on-chip photonic system. Reproduced with permission from Ref. [152]. Copyright: 2025 Springer Nature.

The all-optical non-linear activation scheme is primarily realized through the non-linear optical effects of materials, which can reduce or even avoid the photoelectric conversion process, thus theoretically offering advantages of high speed and low power consumption. However, this approach still faces challenges such as insufficient stability of material non-linear responses and high complexity in process integration. In recent years, two-dimensional materials have been regarded as one of the key materials for achieving all-optical non-linear activation due to their rich optoelectronic non-linear properties. For example, in 2024, Chen *et al*. [148] coupled and integrated two-dimensional MoTe_2_ at the end face of a glass waveguide, leveraging its saturable absorption effect to achieve ultra-broadband all-optical non-linear activation, with an activation threshold as low as 0.94 μW and a response rate of 2.08 THz, and demonstrated classification accuracies of 97.6% on MNIST and 94.6% on CIFAR-10, enabled by the MoTe_2_-based unit shown in Fig. 7c. In the same year, Zhou *et al*. [149] hybrid-integrated a double-slot structure, comprising an Si slot waveguide and a plasmonic slot waveguide, with monolayer graphene. This architecture enhanced light–graphene interaction and leveraged graphene’s saturable absorption property along with its picosecond-scale carrier relaxation time, enabling an ultra-fast all-optical non-linear device with a response time of 93.6 ps, a threshold power of 5.49 mW and a power consumption of 0.51 pJ per activation. Specifically, the ultra-fast Si/graphene optical non-linear activator is shown in Fig. 7d. Besides two-dimensional materials, traditional strong non-linear optical crystals like LN continue to attract attention due to their significant second-order non-linear effects, making them an ideal platform for implementing non-linear activation functions. In 2024, Wang *et al*. [150] constructed a large-scale photonic computing system based on non-linear disordered media, utilizing the second-order non-linear effects of LN nanocrystals to directly provide non-linear transformations, thereby achieving non-linear activation, with a system scale of 27 648 input and 3500 non-linear output nodes, improving classification accuracy by 5.4%–10.9% over linear baselines on sign language digit recognition. Besides this, other material systems have also made advancements in this field. In 2025, Wu *et al*. [151], based on an active III–V semiconductor platform using InGaAsP multiple QWs, extended field-programmable capabilities to the realm of photonic non-linearity for the first time. This architecture employs InGaAsP multiple QW semiconductor thin films as the non-linear medium, synthesizing programmable polynomial functions by controlling carrier dynamics in the semiconductor, achieving programmable non-linear orders up to *N* ≈ 14 and enabling *in situ* training with dynamic reconfiguration. The field-programmable photonic non-linearity architecture is shown in Fig. 7e. In addition to tapping into the potential within traditional and emerging semiconductor materials, harnessing the strengths of different materials through heterogeneous integration represents another crucial technological pathway. In the same year, Liao *et al*. [152], based on a perovskite/Si_3_N_4_ heterogeneous integration platform and utilizing the saturable absorption effect of Bi_2_Te_3_, successfully achieved optical non-linear activation functionality, with a light emitting diode (LED) external quantum efficiency of 21.2%, a PD dark current of 8.3 pA and a response time of 44 ns, achieving a topological model fidelity of 87% and edge-detection accuracy exceeding 85%, using the hetero-integrated perovskite/Si_3_N_4_ on-chip photonic system shown in Fig. 7f.

Beyond the conventional layer-by-layer activation paradigm, PCM-enabled photonic architectures have demonstrated alternative computing modalities that unify weight storage and non-linear thresholding within a single material platform, bypassing the need for separate activation hardware. In 2019, Feldmann *et al*. realized an all-optical spiking neurosynaptic network on an Si_3_N_4_ PIC, in which GST cells serve as both non-volatile synaptic weights and integrate-and-fire neuronal activation elements, interconnected via WDM MRRs [153]. This architecture demonstrated supervised and unsupervised learning as well as four-letter pattern recognition across a 140-element, four-neuron, sixty-synapse integrated circuit (IC). Along a complementary direction, in 2022, Tan *et al*. experimentally demonstrated a backpropagation-free associative learning scheme on an integrated photonic platform, wherein monadic associative learning elements, comprising Si_3_N_4_ directional couplers with a GST phase-change cell, learned to associate two optical inputs through amorphization-induced coupling modulation, achieving repeatable learning–forgetting cycling over 80 cycles with ±0.69% transmission deviation [154]. Scaling to a four-element on-chip network, they performed 1 GHz bit-pattern recognition and image classification using a single associative layer without backpropagation, with a minimum learning energy of 1.8 nJ per element.

### Training strategies for PNNs

The preceding subsections have primarily addressed the inference pipeline of PNNs, where predetermined weights are loaded onto the photonic hardware to perform forward computation. However, the training, which is the process of iteratively optimizing these weights, constitutes an equally critical yet underexplored challenge. In conventional workflows, neural network models are first trained on digital computers and the resulting weight parameters are subsequently transferred to the PIC for inference. This offline approach suffers from a fundamental limitation: the inevitable mismatch between the idealized digital model and the physical hardware, arising from fabrication-induced device variations, thermal drift and crosstalk, can lead to substantial degradation in inference accuracy upon deployment [9]. To bridge this gap, a growing body of research has developed training strategies that directly incorporate the physical characteristics of photonic hardware into the optimization loop, which can be broadly categorized into hybrid training, *in situ* backpropagation and forward-only or gradient-free methods.

Hybrid training methods execute the forward propagation on the actual physical hardware while performing the backward pass digitally, thereby grounding the optimization in the true physical loss landscape. In 2022, Wright *et al*. [155] introduced physics-aware training and experimentally demonstrated it across optical, mechanical and electronic platforms, achieving 97%, 87% and 93% MNIST classification accuracy, respectively, with a hybrid optical–digital architecture boosting accuracy from ∼90% to 97% without a digital output layer. In the same year, Spall *et al*. [156] validated this hybrid paradigm on a free-space coherent optical matrix-vector multiplier with three architectures—a real-valued linear classifier (88.0% MNIST accuracy), a hybrid optoelectronic network with a digital ReLU hidden layer (92.7%) and a complex-valued optical network (92.6%), demonstrating that hybrid training maintains accuracy under 20% static additive noise where purely in silico training degrades from 87.7% to 72.8%.

*In situ* backpropagation goes further by computing gradients directly on the photonic chip, exploiting the reciprocity of optical wave propagation to physically realize the backward pass. The theoretical foundation was established by Hermans *et al*. in 2015 [157], who showed that reciprocity ensures that running signals backward through the same physical medium automatically computes the transposed matrices required by backpropagation, and validated the concept on a simulated EO system achieving ∼30% frame error rate on TIMIT acoustic-phonetic continuous speech corpus (TIMIT) phoneme recognition. Hughes *et al*. in 2018 [158] formalized this insight for MZI-based PNNs via the adjoint variable method, enabling gradient recovery for all phase shifters simultaneously in constant time, with simulations confirming high-fidelity gradient extraction even under 41% scattering loss. This protocol was experimentally realized by Pai *et al*. in 2023 [49] on a foundry-fabricated 6 × 6 Si photonic MZI mesh with a 4 × 4 unitary matrix unit, achieving 93%–96% accuracy on two-dimensional classification tasks with convergence closely tracking the digital baseline. Zhou *et al*. in 2020 [159] extended optical backpropagation to free-space diffractive networks, where a 10-layer network with 150 × 150 neurons per layer achieved 91.96% MNIST accuracy under realistic conditions, rising to 97.48% with optical non-linear layers, and an estimated core-module energy efficiency on the order of 10^11^–10^12^ MAC/(s·W). Most recently, in 2026, Ashtiani *et al*. [160] demonstrated the first fully integrated Si photonic deep neural network capable of end-to-end on-chip backpropagation training, comprising a two-input layer, an eight-neuron hidden layer and a single output neuron, achieving 92.5% classification accuracy that matched the ideal digital model, whereas conventional offline-trained weights transferred to the same chip yielded substantially degraded accuracy due to measured weight deviations up to 0.273 across nominally identical units.

Forward-only and gradient-free methods offer practical alternatives where bidirectional propagation is infeasible. In 2024, Xue *et al*. [161] introduced fully forward mode learning, which replaces the backward pass with a second forward pass by exploiting spatial symmetry and Lorentz reciprocity, and trained networks up to eight layers deep (160 000 neurons per layer) on benchmarks including MNIST, Fashion-MNIST, CIFAR-10 and ImageNet subsets, achieving an energy efficiency of 5.40 × 10^18^ OPS/W in a non-line-of-sight imaging configuration. Cong *et al*. in 2022 [162] demonstrated on-chip training of an Si PIC using a gradient-free bacterial foraging optimization algorithm on a 46-heater SOI device, achieving up to 98.3% test accuracy on Iris classification and ∼94% on simulated MNIST recognition with only 1663 parameters. Complementary to these training paradigms, Bandyopadhyay *et al*. in 2021 [163] proposed a deterministic error-correction protocol for MZI meshes that compensates static beamsplitter fabrication errors via one-time calibration, reducing the average matrix error by more than an order of magnitude in circuits with up to 256 modes and extending fabrication tolerance from ∼3% to beyond 6% while preserving baseline MNIST classification accuracy within 1%.

Overall, training strategies for PNNs have progressed from purely digital offline approaches to increasingly hardware-aware paradigms. Nevertheless, current on-chip training demonstrations remain limited to relatively small-scale networks, and extending these approaches to deep architectures with hundreds of layers, as required for LLM-scale acceleration, demands further advances in signal regeneration, gradient precision and scalable calibration. The co-design of training algorithms with photonic hardware architectures represents a critical frontier for realizing the full potential of PNNs as practical AI accelerators.

## MATERIAL INTEGRATION

The rapid development of photonic integration technologies for on-chip photonic computing has proceeded through several stages, most notably the evolution from hybrid integration (chip-to-chip assembly), to heterogeneous integration (die-to-wafer bonding) and, ultimately, monolithic integration (single-wafer co-fabrication) [10]. For PNNs, although monolithic integration is widely regarded as the ultimate goal, current mainstream approaches are still predominantly based on hybrid and heterogeneous schemes.

### Hybrid integration

Hybrid integration approaches install independently manufactured chips on different substrates separately, and then integrate these chips onto a common platform to achieve the required functions. It provides a potential solution by incorporating disparate photonic technologies into a single integrated system that may not be otherwise compatible in a single fabrication process. Currently, hybrid integration is the most widely used method in the industrial field, and the evolution of the hybrid method has spanned multiple integration schemes, from 2D to 2.5D and 3D architectures [164], as demonstrated in Fig. 8a, with each offering specific trade-offs in interconnect density, signal integrity, thermal management and cost [165].

**Figure 8.**
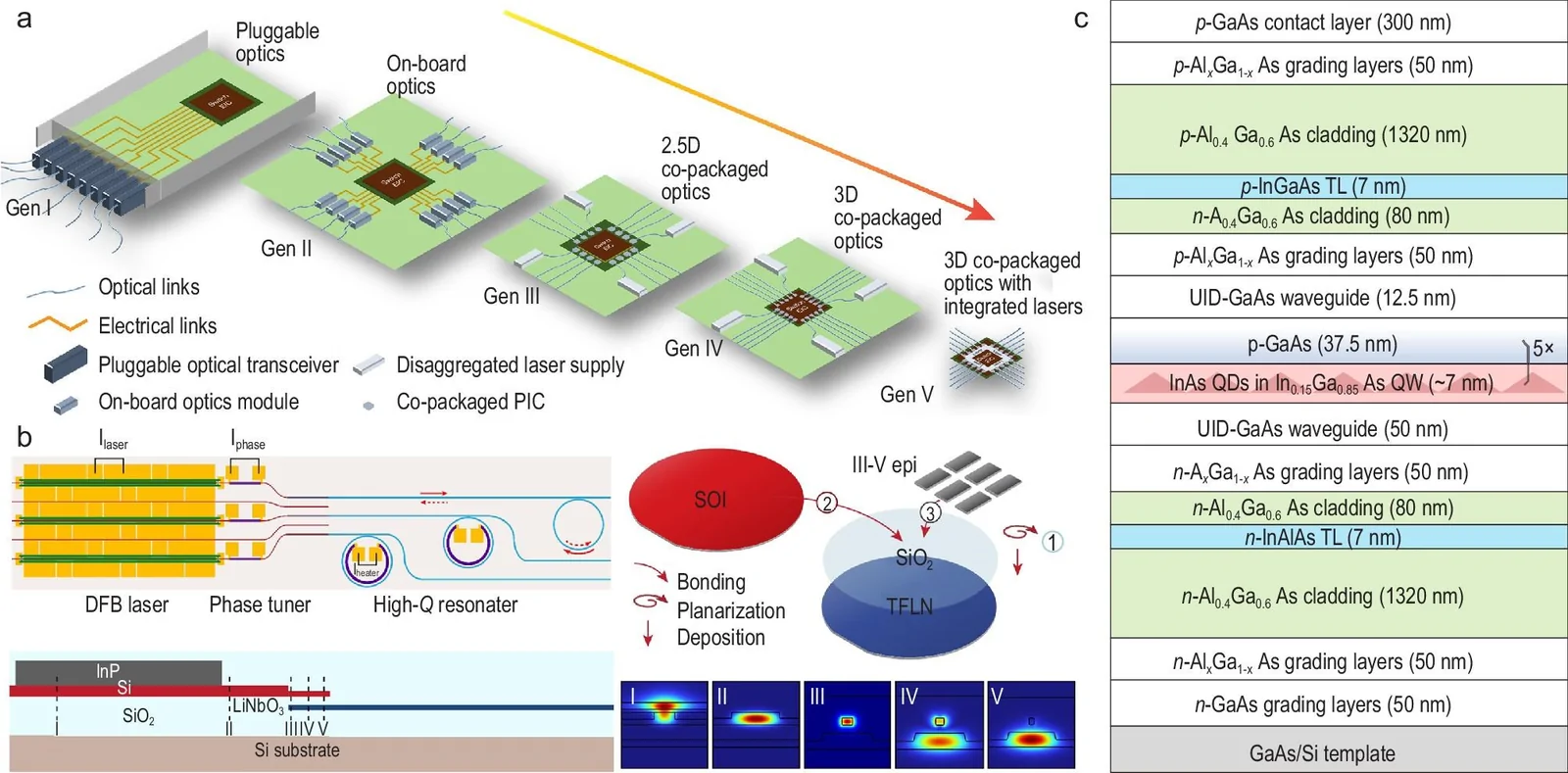
Different photonic integration technologies. (a) Hybrid integration (chip-to-chip assembly). Reproduced from Ref. [164] under a CC BY 4.0 license. (b) Heterogeneous integration (die-to-wafer bonding). Reproduced from Ref. [172] under a CC BY 4.0 license. (c) Monolithic integration (single-wafer co-fabrication). Reproduced from Ref. [175] under the OSA Open Access Publishing Agreement.

2D integration involves placing PICs and EICs side by side on a printed circuit board (PCB), wire bonding and flip-chip bonding remain popular and reliable in 2D hybrid integration [166]. Wire bonding has long been the dominant method for connecting semiconductor dies to package lead frames and circuit boards, particularly in chip-on-board technology, where the die is directly mounted onto the PCB [165]. Flip-chip with C4 bumps and microbumps offer superior connection density compared to peripheral wire bonding [167,168]. The benefits of this method are the ease of packaging technologies, low complexity and cost-effectiveness. However, the drawbacks include significant parasitic inductance, which leads to signal integrity issues and high energy consumption. Additionally, the overall I/O capabilities are restructured due to its pitch limitations and reliance on single-edge connections.

2.5D integration presents a middle ground by mounting both EICs and PICs onto a passive interposer equipped with through-Si vias (TSVs) [169,170]. This method reduces packaging parasitic between EICs and PICs by using microbumps or copper pillars, which facilitate higher I/O connections and enable finer routing for increased bandwidth. While interposers facilitate the inclusion of waveguide layers, improving optical signal redistribution and integrity. However, 2.5D integration is more expensive to develop and fabricate than 2D solutions. Although it outperforms 2D integration, it still entails higher parasitic than 3D architectures, resulting in a net impact on overall performance.

Furthermore, 3D hybrid integration adopts a more sophisticated architecture by stacking EICs directly onto PICs [165]. This is achieved through cutting-edge packaging techniques such as TSV, high-density fan-out, Cu–Cu hybrid bonding or active photonic interposers (where the PIC itself serves as the interposer) [167,169]. Advancements in direct bonding, photolithography and wafer-level alignment have reduced bump pitches from 40–50 μm in early multichip modules to 10–40 μm in current advanced process lines. Furthermore, prototypes are now demonstrating 2 μm die-to-wafer bonding and pitch scaling as low as 400 nm in wafer-to-wafer bonding. In general, 3D integration significantly reduces parasitic and accommodates dense pitch configurations, thereby boosting performance. However, thermal management remains a critical challenge, heat generated by EICs can degrade PIC performance, necessitating robust thermal solutions.

### Heterogeneous integration

Unlike hybrid integration, heterogeneous integration involves bonding dissimilar materials directly onto a common substrate. This integration process is generally performed at the early- to mid-stages of PIC fabrication, such as bonding unpatterned III–V thin films to preprocessed Si [171], Si_3_N_4_ [12] and TFLN [172] PICs. Heterogeneous integration offers the key benefit of monolithic-like functionality, enabling high alignment accuracy and low insertion losses across waveguide transitions. Furthermore, it ensures the reliability of integrated photonic elements while leveraging economies of scale to lower fabrication costs. For instance, by wafer-bonding process, III–V DFB lasers can be integrated on TFLN platforms directly, as shown in Fig. 8b [172]. Heterogeneous integration transitions manufacturing to the wafer level, which enhances integration density and cost-effectiveness by eliminating the need for active alignment. It facilitates the creation of large laser arrays with broad wavelength coverage, aligned to Si photonic waveguides with CMOS-level lithographic precision. Furthermore, because the laser facets are defined within Si rather than III–V materials and are encapsulated by dielectric films, they are shielded from damage and contamination. This structural robustness results in near-lossless optical coupling to Si photonic circuits and best-in-class reliability.

Heterogeneous integration uses various wafer-bonding methods, including polymer, metal and direct bonding. Notably, direct bonding facilitated the breakthrough of the first electrically pumped, Si-based heterogeneous laser, paving the way for commercial Si-based optical transceivers [16]. Sequential wafer bonding has since evolved this technique for multilayer structures. By reducing index mismatches between disparate materials, it expands operational bandwidth and improves performance.

Die and wafer bonding creates permanently bonded stacks by joining a die or wafer to a substrate. This process is essential for integrating large-area films with high material quality, such as crystalline layers that cannot be easily grown on non-native materials. Different wafer-bonding methods have been developed over the years, and they can be categorized depending on the bonding mechanism, namely, without intermediate layer bonding and intermediate layer bonding [173]. For large-scale integration, by using direct wafer-bonding techniques to combine III–V materials with Si substrates, it is possible to effectively circumvent the lattice-mismatch challenges inherent to epitaxial growth.

### Monolithic integration

Monolithic integration embeds photonic components into standard electronic process nodes via epitaxial growth, enabling the coexistence of active photonics and electronics on a single die. This architecture eliminates interface pads and bumps, thereby minimizing parasitics and impedance mismatch while simplifying packaging, providing excellent thermal paths at wafer-scale cost. However, the reliance on legacy CMOS nodes often necessitates a trade-off, resulting in constrained photonic performance and increased energy consumption.

Fabrication processing is the core of monolithic integration, encompassing complex steps such as material deposition, lithographic pattern transfer, etching and surface treatment. The process flow for a single complex device can involve more than 200 individual steps. Through process optimization, key optical functions, including modulation, detection, splitting and coupling, can be integrated on the same Si wafer. Foundational milestones in this field include the realization of the first continuous-wave lasers on miscut Si, alongside the creation of CMOS-compatible substrates. Subsequent innovations in advanced doping and novel active layers have further enhanced device longevity and output power [174]. A pivotal advancement was shifting from QW to QD gain media; this transition significantly bolstered defect tolerance, allowing Si-grown QD lasers to achieve performance levels comparable to traditional QW devices [24]. The laser stack design of representative QD lasers is shown in Fig. 8c [175]. Successful demonstrations now cover sub-milliamp threshold MRRs [176], mode-locked lasers with broad locking ranges [177] and both DFB [178] and tunable lasers [24] exhibiting high side-mode suppression ratios. More recently, efforts have focused on active–passive co-integration on 300 mm patterned Si wafers [174] and optimizing light coupling into Si waveguides [179]. While monolithic integration is not yet as mature as hybrid or heterogeneous methods, the performance gap is rapidly narrowing.

## SYSTEM PACKAGING

To minimize electrical channel loss and signal degradation from copper interconnects, photonic modules are physically migrating closer to the host application-specific integrated circuit (ASIC). This evolution has progressed from traditional pluggable modules to on-board optics (OBO), and now to linear drive pluggable optics (LPO) and co-packaged optics (CPO), where the optics are positioned directly adjacent to the switch or accelerator die. Each distinct architecture, whether pluggable optics, OBO, LPO or CPO, imposes unique and stringent requirements regarding integration density, signal integrity and power efficiency.

### Pluggable optics

Pluggable optics are the most common form of data center optics used today due to their flexibility, scalability and compatibility with various networking devices and standards. Modern ultra-large-scale data centers generally adopt leaf–spine network architectures [5], which achieve efficient interconnection of server clusters through multiple layers of switches and possess features such as strong scalability and low transmission latency. In this architecture, servers are connected to leaf switches, and leaf switches are connected to spine switches through high-speed pluggable optical modules. The mainstream commercial optical modules have evolved from 400 to 800 Gbit/s, and major manufacturers have showcased 1.6 Tbit/s optical module prototypes, while the development of 3.2 Tbit/s modules has also entered a critical stage. As bandwidth demands escalated, traditional pluggable optical modules encountered severe limitations regarding power consumption and signal integrity. As data rates escalate, thermal management becomes increasingly complex, with heat dissipation emerging as a fundamental barrier to reliable device operation. To mitigate these thermal constraints, the industry is actively pursuing alternative packaging architectures designed to shorten electrical signal paths and drastically minimize insertion losses, thereby reducing the overall power required to drive the signal. However, pluggable optics continue to evolve alongside new architectures. Innovations such as coherent optics are extending the capabilities of traditional form factors, to meet the massive scaling requirements of modern AI infrastructure [180].

### OBO

OBO integrates optical components directly onto a PCB to convert electrical signals to light signals for high-speed data transmission. This integration shortens the electrical signal path between the transceivers and switch ASICs, which reduces signal loss and power consumption and allows for higher-density connections compared to traditional pluggable modules. Early implementations of OBO were successfully demonstrated in high-performance computing environments [181], leading to standardized modules capable of 800 Gb/s. However, it required a large footprint on the main circuit board without delivering a substantial reduction in channel length. Because of these limitations, widespread adoption never materialized, and the industry’s focus has largely shifted to the superior integration offered by CPO.

### LPO

LPO is a type of optical transceiver that eliminates the digital signal processor (DSP) chip to reduce power consumption, cost and latency in high-speed data center connections. By moving the processing functions to the host system, LPO modules are more power-efficient and suitable for short-reach applications like server-to-switch connections within a data center, LPO reduces latency and lowers power consumption by ∼40% [182]. By design, LPO offers a scalable path to reconciling high data rates with low power consumption for pluggable modules, while CPO enables direct integration of photonics onto the switch IC, thereby eliminating the need for a standalone module. Although CPO is becoming increasingly popular, LPO is seen as a natural evolutionary path for pluggables, offering lower risk compared to CPO, especially in terms of reliability. From a serviceability perspective, LPO offers a distinct advantage by utilizing hot-swappable modules, whereas the tight integration of CPO makes replacement difficult. LPO acts as a strategic alternative, leveraging the linear drive approach to optimize power, cost and latency without sacrificing the operational flexibility of pluggable modules.

### CPO

CPO emerged as critical solutions to overcome severe limitations regarding power consumption, signal integrity and thermal management in high-speed signal transmission. The integration burdens CPO with higher reliability demands, driven by greater thermal stress, system complexity and overall cost. However, CPO achieves data rates and power efficiency that pluggable optics struggle to match. Broadcom pioneered this space in 2021 with the first 25.6 Tbit/s CPO switch, laying the groundwork for future exploration. Building on this foundation, they subsequently introduced a 51.2 Tbit/s CPO switch featuring a single-channel rate of 100 Gbit/s, followed in 2025 by a third-generation architecture utilizing single-wave 200 Gbit/s technology [183]. Similarly, NVIDIA Quantum-X series CPO switch, released in 2025, leverages advanced 3D heterogeneous packaging to achieve a total switching capacity of 115.2 Tbit/s [184]. This device integrates four TSMC 4 nm chips with 18 Si photonic engines, each housing eight MRMs capable of 200 Gbit/s signal modulation. Compared to traditional pluggable solutions, the Quantum-X delivers substantial performance gains: 3.5 times greater energy efficiency, 63 times better signal integrity, a 10-fold increase in network reliability and 1.3 times faster deployment. Furthermore, by directly 3D-packaging the photon engines with the ASIC and utilizing liquid cooling, the switch effectively manages high-density thermal loads while reducing the reliance on external lasers and optical fibers.

System packaging is important for PNNs not only because it enables different levels of optoelectronic integration, but also because it will affect the upper bound of system performance. Specifically, the packaging scheme determines the interconnect distance and interface form between the ASIC and the photonic computing core, thereby influencing link loss, signal integrity, latency, bandwidth density and power consumption [164]. In general, the evolution from pluggable optics, OBO and LPO to CPO is accompanied by a progressive increase in integration level and a reduction in the electrical path length, which is beneficial for improving interconnect bandwidth. The enhanced signal integrity associated with higher-level packaging can also reduce reliance on DSP, thereby lowering the energy per bit. One of the main reasons CPO has attracted broad attention is that, by placing the optical engine as close as possible to the ASIC, it can increase system bandwidth while reducing the energy consumption per bit [183]. For PNNs, the resulting optimization of I/O in bandwidth and power consumption at the packaging level will affect the computational efficiency and achievable scale of the overall system, beyond the communication link itself. However, it should also be noted that, many photonic computing components in PNNs, especially interferometric and resonator structures, are highly sensitive to thermal drift, crosstalk and fabrication variations. Therefore, although dense packaging can provide higher bandwidth density and better energy efficiency, it may also increase the complexity of thermal management and system maintenance. From the perspective of system implementation, pluggable optics and OBO are more favorable for modular design, testing and serviceability, whereas CPO is more suitable for future large-scale PNN systems requiring extreme integration density; LPO may be regarded as an intermediate solution that preserves the pluggable ecosystem while optimizing link power consumption and complexity. Therefore, for PNN system design, the choice of packaging strategy is essentially a trade-off among bandwidth density, energy efficiency, thermal stability, scalability and serviceability.

Beyond enabling high-level integration and packaging, recent advances in system-level optoelectronic technologies have also begun to broaden the application scope of PNNs toward more realistic AI workloads. Accordingly, the application focus of PNNs is moving beyond early proof-of-concept tasks centered on small-scale classification toward directions that are more closely aligned with current AI demands. In generative AI, recent studies have not only demonstrated optical generative models, but have also proposed dedicated photonic accelerators for generative adversarial networks, targeting representative generative operators such as transposed convolution and instance normalization [185–187]. These developments indicate that photonic computing is extending from conventional discriminative tasks to generative modeling. For more advanced AI models, recent work has demonstrated a universal photonic AI processor capable of executing models such as ResNet and BERT, suggesting that the target of photonic AI hardware is shifting from simplified benchmarks toward more practical neural network workloads [108]. In addition, with progress in large-scale integration and advanced packaging, a photonic matrix accelerator integrating more than 16 000 photonic components has been reported for high-throughput, low-latency matrix computation, reflecting a transition from isolated device demonstrations to more system-relevant AI acceleration platforms [60]. Therefore, emerging research directions include acceleration of selected operators in generative models, high-throughput linear computation for advanced neural network models and system-level photonic AI acceleration under high-bandwidth and low-latency constraints. Driven by the rapid growth of large-model and generative-AI workloads, these application directions are under active exploration, and their broader deployment will further benefit from progress in advanced system packaging and large-scale optoelectronic integration.

## KEY TECHNICAL BOTTLENECKS AND SOLUTION PATHWAYS

### Optical loss

As photonic computing cores scale to accommodate larger matrix dimensions and deeper network architectures, accumulated optical propagation loss emerges as a fundamental bottleneck constraining system performance. In cascaded interferometric meshes such as MZI arrays, each additional stage introduces incremental insertion loss from waveguide propagation, directional coupler imperfections and phase-shifter absorption, which collectively attenuate the signal and degrade SNR at the output PDs [7,83]. For WDM architectures relying on MRR arrays, the aggregate through-port and drop-port losses across multiple resonators similarly limit the number of wavelength channels that can be practically cascaded while maintaining adequate computational precision [8]. This cumulative loss imposes a practical ceiling on both the achievable network depth and the matrix dimensionality, directly restricting the computational capacity of on-chip PNNs.

To address this challenge, recent efforts have pursued solutions at both the material platform and the architectural levels. At the device level, advances in ultra-low-loss waveguide fabrication have substantially expanded the optical loss budget available for large-scale integration. Notably, Bose *et al*. demonstrated anneal-free Si_3_N_4_ PICs achieving propagation losses as low as 1.77 dB/m with intrinsic *Q*-factors of 14.9 million at a maximum process temperature of only 250°C [188], thereby enabling heterogeneous integration with temperature-sensitive III–V and organic materials without compromising waveguide quality. More recently, Chen *et al*. reported Ge–silicate PICs achieving fiber-like losses of 0.08 dB/m at 1064 nm with *Q*-factors exceeding 180 million, representing a significant step toward eliminating propagation loss as a limiting factor for deep on-chip PNNs [189]. At the architectural level, complementary strategies have emerged to mitigate the impact of residual optical loss through topology optimization. Fldzhyan *et al*. introduced circular beam-splitter mesh architectures that implement arbitrary *N* × *N* non-unitary matrix transformations with only *N* + 2 phase-shift layers, substantially reducing the required optical depth compared to conventional singular value decomposition (SVD)-based decompositions and thereby alleviating cumulative loss [190]. Furthermore, Zhou *et al*. demonstrated that single-layer photonic computing (SLiM) with on-chip perturbation injection can construct neural networks exceeding 100 layers while maintaining robust accuracy, offering an alternative paradigm that fundamentally circumvents the error accumulation inherent in conventional multilayer photonic architectures [191].

Despite these advances, a gap remains between the maturity of device-level and architecture-level solutions. Ultra-low-loss waveguide platforms have progressed to experimental validation on foundry-compatible processes and are approaching practical deployment readiness. In contrast, architecture-level strategies that reduce effective optical depth or bypass cascaded loss accumulation, while theoretically compelling, have thus far been demonstrated only on relatively simple classification benchmarks, and their effectiveness on complex, large-scale AI workloads remains to be established. A further challenge common to both approaches is that standalone waveguide loss figures do not capture the total insertion loss budget of a complete computing core, which must additionally account for active phase-tuning elements, waveguide crossings and fiber-to-chip interfaces. Closing this gap will likely require the co-optimization of ultra-low-loss material platforms, loss-tolerant network topologies and on-chip optical amplification strategies.

### Thermal instability and crosstalk

Thermal instability and crosstalk constitute another critical bottleneck that intensifies as photonic computing cores scale to larger dimensions. In MZI-based architectures, thermo-optic phase shifters are the predominant tuning mechanism, yet their operation inherently generates localized heating that propagates to adjacent interferometric arms and neighboring devices, inducing parasitic phase errors that compromise matrix computation fidelity [83]. This thermal crosstalk effect becomes increasingly severe as device pitch decreases to improve integration density, creating a fundamental tension between scalability and computational accuracy. For MRR-based WDM architectures, the challenge is equally pronounced: the resonance wavelength of Si MRRs shifts at ∼80 pm/°C, meaning that even modest ambient temperature fluctuations or heat dissipated by co-integrated electronics can detune the operating point, necessitating continuous thermal stabilization loops whose aggregate static power consumption can dominate the system energy budget as array sizes grow [13].

Recent research has addressed this bottleneck through multiple complementary strategies. At the device level, Ning *et al*. introduced a closed heat shield structure based on scattering-cancellation theory from thermal metamaterials, achieving temperature reductions of up to 50% at neighboring device locations while simultaneously halving the thermal response time without degrading heating efficiency [192]. As an alternative to active thermal management, Lederman *et al*. demonstrated a passive thermal desensitization technique that exploits intrinsic self-heating non-linear feedback in Si MRRs, achieving 49% reduction in thermal crosstalk sensitivity with a latency of only 1 μs, ∼500× faster than conventional active stabilization schemes, while preserving weighting accuracy without external thermal controllers [193]. From a more fundamental perspective, eliminating the reliance on volatile thermo-optic tuning altogether represents a promising long-term direction. Tossoun *et al*. demonstrated a heterogeneously integrated memresonator combining a metal-oxide memristor with an Si MRR, achieving non-volatile photonic phase tuning with sub-picojoule switching energy and 12-h retention, thereby removing the need for static thermal tuning power consumption during inference [194]. Furthermore, to enable systematic design and optimization under thermal effects, Marchisio *et al*. developed a comprehensive physics-informed circuit model for MZI-based meshed topologies that captures thermal crosstalk and fabrication variations, enabling rapid offline programming and quantitative robustness assessment under realistic operating conditions [195].

Nevertheless, achieving thermal stability across large-scale arrays comprising hundreds to thousands of active elements remains an open challenge, and the proposed solutions vary considerably in both maturity and transformative potential. Thermal isolation structures and physics-informed modeling tools are the most readily deployable, as they can be incorporated into existing design and fabrication flows with moderate engineering effort. Passive self-stabilization techniques offer attractive low-latency alternatives that avoid the complexity of active feedback loops. The most transformative long-term direction lies in non-volatile tuning mechanisms that fundamentally eliminate static thermal power consumption; however, current implementations face limitations in retention time and cycling endurance that preclude reliable long-term deployment. Ultimately, no single strategy is likely to be sufficient in isolation, and practical high-density PNN accelerators will demand the co-optimization of these complementary approaches, potentially in conjunction with advanced co-packaging solutions that spatially separate heat-generating electronics from thermally sensitive PICs.

### All-optical non-linear activation

As reviewed in the section entitled ‘Nonlinear activation functions in PNNs’, significant progress has been made in developing all-optical non-linear activation functions based on diverse material platforms, including two-dimensional materials, LN nanocrystals, III–V semiconductors and heterogeneously integrated systems. These demonstrations have expanded the available non-linear mechanisms from saturable absorption and second-order effects to field-programmable carrier dynamics. However, despite this growing material diversity, several fundamental challenges remain that limit the practical deployment of all-optical non-linear activation in large-scale PNNs.

A first challenge concerns the trade-off between non-linear response strength, operating power and integration compatibility. Many demonstrated all-optical non-linearities require relatively high optical power densities to trigger sufficient response, which conflicts with the attenuated signal levels typically encountered after cascaded linear stages in on-chip PNNs. Recent work has begun to address this gap from different angles. Slinkov *et al*. demonstrated an all-optical activation function based on stimulated Brillouin scattering in optical fibers, which provides net gain up to 21.9 dB to compensate upstream insertion losses while delivering tunable activation shapes with inherent frequency selectivity compatible with WDM architectures [196]. Zhong *et al*. demonstrated reconfigurable phase-relevant activation functions using graphene/Si heterojunctions integrated in MRRs, achieving modulation at only 1 V with greater than 12 dB extinction ratio and 7%–8% accuracy improvement through complex-valued processing on MNIST and CIFAR-10 [59]. Tong *et al*. exploited synergistic non-linear absorption transitions in bare MoS_2_ arrays, realizing programmable activation with ∼5.47 ps operating time per cell and picojoule-level energy consumption [197]. These results illustrate that emerging material systems can provide the requisite non-linear characteristics at progressively lower power thresholds, though on-chip integration of fiber-based and free-space approaches remains a significant engineering challenge.

Despite these encouraging single-device demonstrations, significant barriers remain before all-optical non-linear activation can be considered viable for practical PNN deployment. At the integration level, the demonstrated material-based non-linearities generally lack the fabrication maturity and long-term operational stability required for reliable incorporation within complex PICs on standard platforms. More fundamentally, even if individual non-linear units achieve satisfactory performance, a critical open question is whether they can be cascaded across deep network architectures without prohibitive degradation. Current experimental validations are almost exclusively confined to single-stage or few-layer configurations, leaving it unclear whether adequate signal fidelity, SNR and dynamic range can be sustained through 10 or more successive non-linear stages without intermediate signal regeneration. Until this cascadability gap is addressed alongside the development of standardized CMOS-compatible integration processes, all-optical non-linear activation will likely remain confined to shallow network topologies or hybrid architectures where electronic non-linearities handle the depth-scaling burden.

### Scalability and integration density

Scaling PNNs from laboratory-scale demonstrations to computing cores capable of handling practical AI workloads presents multifaceted challenges spanning device density, system integration, packaging and manufacturing yield. Current experimental PNN demonstrations are typically limited to matrix dimensions on the order of tens to hundreds, whereas competitive AI accelerators routinely process matrices with dimensions of thousands or more. For MZI-based architectures, the O(*N*^2^) scaling of component count with matrix dimension, combined with the millimeter-scale footprint of individual interferometric units, imposes stringent area constraints that limit practical on-chip matrix sizes [7]. MRR-based WDM architectures alleviate this footprint challenge through wavelength-domain parallelism, yet they face their own scaling barriers related to the finite FSR, interchannel crosstalk and the need for individual thermal stabilization of each resonator as array sizes grow.

Recent advances have explored complementary strategies to overcome these scaling limitations across multiple levels. At the device level, inverse-design methodologies offer a promising route to dramatically increase integration density by exploiting subwavelength structural optimization. Sved *et al*. demonstrated inverse-designed PNN accelerators achieving ∼400 million trainable parameters per mm^2^, with experimentally validated on-chip MNIST and MedNIST classification within footprints as compact as 20 × 20 μm^2^, representing orders-of-magnitude improvements in parameter density over conventional interferometric approaches [198]. At the system level, Hua *et al*. demonstrated that current commercial Si photonic platforms can support the integration of over 16 000 photonic components with 2.5D hybrid packaging, achieving 64 × 64 optical matrix operations at 1 GHz clock frequency and delivering two orders of magnitude latency improvement over electronic processors on Ising solver benchmarks [60]. To further extend scaling beyond single-chip limits, advanced packaging architectures based on interchip optical couplers and photonic chiplets provide a viable path toward petabit-per-second-scale bandwidth. Weninger *et al*. reviewed the development of waveguide-to-waveguide couplers enabling 3D integrated photonic packaging, demonstrating that flip-chip evanescent coupling architectures can achieve optical I/O densities exceeding 100 couplers/mm, thereby enabling optical fan-out from computing dies to fiber arrays that would be infeasible with conventional edge-coupling approaches [199].

As PICs scale to incorporate thousands of active elements, fabrication process variations become a dominant factor that compounds the aforementioned integration challenges. Xing *et al*. developed a hierarchical spatial variability model for 200 mm Si photonic wafers, revealing that systematic intra-wafer variations in waveguide width and thickness, driven by lithographic exposure dose and chemical mechanical polishing non-uniformity, are the primary contributors to device parameter spread [200]. Such process-induced variability directly impacts the programming accuracy of large-scale MZI and MRR arrays, creating a feedback loop in which scaling amplifies the very imperfections that degrade computational fidelity. Among the various scaling strategies, integration on commercial Si photonic platforms represents the most mature and nearest-term path, as demonstrated by successful fabrication of large-scale circuits. Inverse-designed ultra-compact architectures and chiplet-based multidie assemblies offer substantially higher density ceilings, but they remain at earlier stages of development where design automation tools, packaging reliability and yield management must catch up before production-grade deployment becomes feasible. Bridging these gaps will require the co-development of variation-aware network architectures, statistical process control methodologies and robust multidie assembly processes.

## CONCLUSIONS

In summary, this review systematically summarized the evolving landscape of emerging integrated PNNs, covering the critical path from hardware device foundations to system-level packaging. As the exponential growth in AI scaling increasingly conflicts with the physical limitations of electronic interconnects and memory bandwidth, photonics has emerged not merely as a transmission carrier but as a potent computing platform. For on-chip PNNs, high-performance passive and active devices constitute the physical basis, while diverse network architectural schemes define the efficiency and throughput of the computing core. Furthermore, the transition from mature hybrid integration to rapidly developing heterogeneous and monolithic integration platforms, coupled with the paradigm shift toward advanced system packaging, illustrates a clear industry path aimed at addressing the core data transmission bottlenecks of modern AI workloads.

However, the evolution from laboratory prototypes to commercial AI accelerators faces challenges that necessitate interdisciplinary solutions. For example, as photonic computing cores scale up to accommodate larger matrix dimensions, accumulated optical loss, crosstalk and thermal instability impose strict requirements on fabrication precision and active control mechanisms. Additionally, while linear operations can be performed in optics domain with high efficiency, the realization of ultra-fast, low-power and all-optical non-linear activation functions remains a complex frontier. In the future, the developments in PNNs are likely defined by a functional strategy of deep optoelectronic fusion, in which photonics takes charge of large-scale linear algebra tasks and interconnects, while electronics maintains dominance in non-linear logic, storage and complex control flow, thus achieving an optimal balance between performance and cost.

From a system architecture perspective, PNNs are not anticipated to replace general-purpose electronic processors, but rather to serve as domain-specific heterogeneous accelerators within the broader electronic AI computing ecosystem, analogous to the role that GPUs and TPUs currently play relative to CPUs. In this paradigm, PNNs would be responsible for accelerating the most computationally intensive and latency-sensitive linear algebraic operations, such as large-scale MVM in transformer attention layers and convolutional kernels, while electronic processors continue to handle non-linear activation, normalization, memory management and complex control flow. This functional partitioning leverages the complementary strengths of both domains: the ultra-high bandwidth and energy-efficient parallelism of photonics for linear operations, and the flexibility, precision and programmability of electronics for other functions. Realizing this vision requires not only the maturation of photonic hardware but also the development of standardized optoelectronic interfaces, compiler toolchains capable of mapping neural network graphs onto hybrid photonic-electronic architectures and co-designed software stacks that can dynamically allocate workloads between optical and electronic resources based on precision requirements and latency constraints.

Beyond technical challenges, several economic and industrialization factors will critically influence the commercialization trajectory of PNNs. A foremost consideration is manufacturing cost: while Si photonic fabrication benefits from leveraging existing CMOS foundry infrastructure, PNN-specific requirements, such as ultra-low-loss waveguides, heterogeneously integrated III–V gain elements and non-volatile phase-change tuning materials, often demand specialized process modules that are not yet available on standard multiproject wafer runs, thereby limiting production volume and increasing per-unit cost. Packaging and testing represent another significant cost driver, as the precision alignment of fiber arrays, the co-integration of photonic and electronic dies and the calibration of large-scale programmable meshes all require procedures that are substantially more complex and time-consuming than those for purely electronic chips. Furthermore, the absence of standardized benchmarking frameworks for photonic AI accelerators makes it difficult to perform fair performance comparisons with mature electronic counterparts, hindering adoption by the broader AI hardware community. Establishing industry-wide metrics that account for the unique characteristics of photonic computing, including effective bit precision under realistic noise conditions, energy per MAC operation inclusive of all peripheral electronics and end-to-end inference latency, will be essential for enabling transparent evaluation and accelerating commercialization.

Looking forward, the further performance enhancement of emerging integrated PNNs will benefit significantly from the convergence of advanced material platforms and computing core designs. The exploration of novel photonic materials, such as TFLN and Si_3_N_4_, is expected to unlock new capabilities in modulation speed, integration density, transmission loss and non-volatile photonic memory, potentially facilitating the realization of more practical and versatile PNNs. As manufacturing processes standardize and advanced packaging technologies become mainstream, on-chip PNNs based on optoelectronic integration platforms are positioned to evolve from standalone demonstrators into co-integrated accelerator modules within next-generation AI infrastructure, offering a transformative path toward sustainable high-performance computing in the post-Moore era.
